# Micropopulation mapping of the mouse parafascicular nucleus connections reveals diverse input–output motifs

**DOI:** 10.3389/fnana.2023.1305500

**Published:** 2024-01-08

**Authors:** Enrique Gonzalo-Martín, Carmen Alonso-Martínez, Lucía Prensa Sepúlveda, Francisco Clasca

**Affiliations:** Department of Anatomy and Neuroscience, Autónoma de Madrid University, Madrid, Spain

**Keywords:** thalamostriatal, thalamocortical, corticothalamic, basal ganglia, motor cortex, cortical layer 5

## Abstract

**Introduction:**

In primates, including humans, the centromedian/parafascicular (CM-Pf) complex is a key thalamic node of the basal ganglia system. Deep brain stimulation in CM-Pf has been applied for the treatment of motor disorders such as Parkinson’s disease or Tourette syndrome. Rodents have become widely used models for the study of the cellular and genetic mechanisms of these and other motor disorders. However, the equivalence between the primate CM-Pf and the nucleus regarded as analogous in rodents (Parafascicular, Pf) remains unclear.

**Methods:**

Here, we analyzed the neurochemical architecture and carried out a brain-wide mapping of the input–output motifs in the mouse Pf at micropopulation level using anterograde and retrograde labeling methods. Specifically, we mapped and quantified the sources of cortical and subcortical input to different Pf subregions, and mapped and compared the distribution and terminal structure of their axons.

**Results:**

We found that projections to Pf arise predominantly (>75%) from the cerebral cortex, with an unusually strong (>45%) Layer 5b component, which is, in part, contralateral. The intermediate layers of the superior colliculus are the main subcortical input source to Pf. On its output side, Pf neuron axons predominantly innervate the striatum. In a sparser fashion, they innervate other basal ganglia nuclei, including the subthalamic nucleus (STN), and the cerebral cortex. Differences are evident between the lateral and medial portions of Pf, both in chemoarchitecture and in connectivity. Lateral Pf axons innervate territories of the striatum, STN and cortex involved in the sensorimotor control of different parts of the contralateral hemibody. In contrast, the mediodorsal portion of Pf innervates oculomotor-limbic territories in the above three structures.

**Discussion:**

Our data thus indicate that the mouse Pf consists of several neurochemically and connectively distinct domains whose global organization bears a marked similarity to that described in the primate CM-Pf complex.

## Introduction

Several nuclei of the thalamus are major nodes of the motor system. Neurons in these nuclei integrate convergent inputs from different motor-related subcortical and cortical cell populations and target the dorsal striatum and motor cortex, often simultaneously, through branched axons. In the striatum, thalamic inputs are important for motor sequence selection and switching (Matsumoto et al., 2001; Minamimoto and Kimura, 2002; Minamimoto et al., 2005, 2009; Redgrave et al., 2010; Alloway et al., 2014; Martel and Galvan, 2022). In the motor cortex, thalamic inputs have been shown to support persistent activity during planning, execution and learning of rapid and precise motor behaviors (Gao et al., 2018; Guo et al., 2018; Catanese and Jaeger, 2021; Inagaki et al., 2022).

In anthropoid primates, the centromedian (CM) and parafascicular (Pf) nuclei constitute the main source of thalamostriatal projections (Sadikot and Rymar, 2009; Smith et al., 2014). These two nuclei are often regarded as a single complex (“CM-Pf”), as the cytoarchitectonic border between them is not sharp. Interestingly, these two nuclei share several cellular, physiological, and circuit features that are unlike those of all other nuclei of the thalamus [reviewed in Jones (2007); Smith et al. (2014)]. The primate CM receives mainly medial globus pallidus (GPi) inputs and projects massively to the sensorimotor striatum, and, more weakly, to the premotor and motor cortices. The Pf receives mainly substantia nigra pars reticulata inputs (SNr) inputs and projects to the limbic and associative striatum and, weaklier, to the prefrontal and limbic cortices. Additionally, differences between CM and Pf in neurochemical markers such as acetylcholinesterase (Jones, 2007; Paxinos et al., 2012), calbindin (Sadikot et al., 1992; Paxinos et al., 2012), or glycine transporter type 2 (GlyT2; Giber et al., 2015) have been described in primates. Neuronal degeneration has been reported to occur specifically in the CM-Pf complex in basal ganglia disorders such as progressive supranuclear palsy and Parkinson’s disease (Henderson et al., 2000a,b). Deep brain stimulation has been applied in the human CM-Pf to treat Tourette syndrome-related symptoms, dyskinesia, parkinsonian tremor, and dystonia [Testini et al., 2016; Picillo et al., 2017; reviews in Smith et al. (2014) and Baumgartner et al. (2022)].

Rodents are increasingly important models in the study of the cellular, genetic, and neural circuit mechanisms of brain motor systems function and disease. Based on the fragmentary data available, a single cell mass in the rodent thalamus, the parafascicular nucleus (Pf) is traditionally regarded as equivalent to the primate CM-Pf complex (Jones, 2007; Galvan and Smith, 2011). Because thalamic nuclei are essentially defined by their inputs and outputs (Halassa and Sherman, 2019; Acsády, 2022) a comprehensive mapping of the rodent Pf connectivity might provide crucial evidence to gauge the similarities/differences with the primate CM-Pf.

Published studies on rodent Pf connections (Berendse and Groenewegen, 1990; Krout and Loewy, 2000a,b; Krout et al., 2001, 2002; Van der Werf et al., 2002; Kita et al., 2016; Mandelbaum et al., 2019; Foster et al., 2021) together indicate that neurons situated laterally or medially within Pf differ in their connectivity, revealing a general resemblance to the primate CM and Pf, respectively (Galvan and Smith, 2011). However, important questions such as the prevalence of the various cortical (layer 5; L5 and layer 6; L6) and subcortical input systems remains unclear. Likewise, the arborization structure of Pf axons in their various cortical and subcortical targets has not yet been analyzed and directly compared. Precise data on these basic circuit parameters are key for building biologically accurate models of Pf neuron computations and their contribution to motor functions.

In the present study, we set out to (a) chart and quantify the sources of input to the mouse Pf and their distribution within the nucleus, (b) measure and compare the terminal arborization structure of axons originated from different Pf subregions in the cortex, the dorsal striatum (CPu) and the subthalamic nucleus (STN); and (c) compare the input–output patterns of mouse Pf with those reported for the CM-Pf complex in anthropoid primates.

## Materials and methods

### Animals

A total of 42 adult (90–150 days old, 25–32 g in weight) male C57BL/6 mice were used for the experiments reported in this study. Mice were housed under standard colony conditions with food and water *ad libitum* under a 12-h light/dark cycle. All procedures were conducted at the Autónoma de Madrid University under protocols approved by our University Ethics committee and the competent Regional Government agency (PROEX 179.3/21), in accordance with the European Community Council Directive 2010/63/UE. Taking advantage of the absence of contralateral projections from the dorsal thalamus, to minimize the number of animals required, the BDA-only experiments (see below) were simultaneously performed on both cerebral hemispheres.

### Anesthetic procedures

All surgical procedures were conducted under isoflurane anesthesia (1–2% in oxygen) following induction with a combination of ketamine (0.075 mg/g body weight, i.p.) and xylazine (0.02 mg/g body weight, i.p.). During surgery, the appropriate level of anesthesia was monitored by regularly testing the absence of tail pinch withdrawal reflex while maintaining spontaneous regular breathing. Buprenorphine hydrochloride (0.075 mg/kg body weight, s.c.) was administered for post-surgical analgesia, complemented with ibuprofen (0.012 mg/mL) added to water bottles for the duration of the survival period. At the time of sacrifice, animals received a lethal dose of sodium pentobarbital (0.08 mg/g body weight, i.p.) before perfusion (see below).

### Surgical procedures for axonal tracer injections

For connection tracing experiments, the anesthetized animals were positioned in a stereotaxic frame (David Kopf Instruments, Tujunga, CA, United States) and placed on a water-heated pad at 37°C. The midline of the scalp was then sectioned and retracted, and a small craniotomy was opened.

In experiments aimed at labeling the axons originated in particular domains of the Pf nucleus, borosilicate glass micropipettes (1 mm outer diameter with internal glass filament; 10–20 μm of inner tip diameter; World Precision Instruments, Sarasota, FL, United States) were loaded with a 3% solution of lysine-fixable biotinylated dextran amine (BDA) of 10,000 MW (Invitrogen, Carlsbad, CA, United States) in 0.01 M phosphate buffer (PB; pH 7.4). In experiments aimed at simultaneously revealing both the Pf input sources and its output pathways, BDA (3%) was mixed with *Vibrio cholerae* toxin subunit B (CTb, 1%; List Biological Laboratories, CA, United States) in PB.

Pipettes were stereotaxically positioned following coordinates according to the Paxinos and Franklin (2019) mouse atlas. A positive current of 400–600 nA (1 s on/off cycles) was applied for 30–40 min using a Midgard Current Source (Stoelting Co., Wood Dale, IL, USA). The micropipette was left in place for 10 min before removal and wound closure. At the end of the surgery, isoflurane was interrupted, and the animals were allowed to recover before being finally returned to their cages. Animals were left to survive for 7 days after the injection to allow for axonal transport.

### Tissue processing for axonal tracing experiments

Following sacrifice, animals were perfused transcardially with 30 mL of saline, followed by 100 mL of 4% paraformaldehyde (PFA; diluted in 0.1 M PB, pH 7.4) for 8 min. Brains were then removed from the skull and postfixed by immersion for 2 h at 4°C in the same fixative. Subsequently, brains were cryoprotected by soaking in 30% sucrose (0.1 M PB, 4°C, 48 h). Serial 50 μm-thick coronal sections were obtained on a freezing microtome (SM 2400; Leica, Germany).

In experiments aimed at anterogradely labeling thalamofugal axons, all sections were incubated to reveal BDA. Peroxidase activity was blocked by incubation in PB-buffered H_2_O_2_ for 15 min, and sections were then incubated in avidin-biotin-peroxidase complex (ABC, 1:100; Vectastain Elite, Vector Laboratories) diluted in PB overnight at 4°C. After washing in PB, labeling was visualized using the glucose oxidase-3–3-diaminobenzidine (DAB) nickel sulfate-enhanced method (Shu et al., 1988). To delineate the striosome compartment of the caudate-putamen, we applied immunohistochemistry for μ-opioid receptor (MOR) as a counterstain.

In experiments combining CTb and BDA, BDA was first revealed as above; subsequently, the sections underwent overnight incubation in an anti-CTb rabbit antiserum (1:500) at room temperature (RT), rinsed and then incubated in anti-rabbit goat antiserum (1:500; for 2 h at RT). Finally, sections were incubated in ABC (2 h; RT), and then in glucose oxidase and DAB without nickel, to reveal the CTb. Thionin counterstain was applied as an aid for the cytoarchitectonic localization of the labeling.

Sections were serially mounted onto gelatin-coated glass slides, air-dried, dehydrated in graded ethanol, defatted in xylene and coverslipped with DePeX (Serva Electrophoresis, Germany).

### Tissue stainings for nuclei delineation

Eight mice brains were used for a multilabeling assessment of the Pf borders. These brains were cut either in the coronal (*n* = 4), sagittal (*n* = 2), or horizontal (*n* = 2) planes. Adjacent series of sections were each stained with cresyl violet (Nissl), with Cytochrome C-oxidase (CytOx; Wong-Riley et al., 1978) or acetylcholinesterase (AChE; Geneser-Jensen and Blackstad, 1971) histochemistry, or with calbindin (CB) or glycine transporter type 2 (GlyT2) immunohistochemistry using commercial antibodies and standard protocols (Supplementary Table S1). Sections were finally mounted and coverslipped as above.

### Mapping of injection sites and axonal transport labeling

The location of each BDA or CTb injection site was reconstructed from serial coronal sections. Digital images of Thionin- or MOR-counterstained sections were taken on a Nikon Eclipse 50i microscope and 4-10X objectives. Under brightfield microscopy, the microiontophoretic BDA deposits appeared as a black compact mass of cell somata and neurites. In the CTb experiments, the injection site was covered by a brownish traslucent precipitate.

To analyze the striatal labeling, in each valid case (*n* = 37) a complete series of BDA-stained + MOR-counterstained sections was examined. Axonal arborizations and striosomes were then digitally traced at high magnification on five representative section levels using a Neurolucida platform (MBF Biosciences) mounted on a Nikon 80i microscope with 20-40X bright-field optics. To analyze the cortical labeling, axons were examined and plotted onto an unfolded map of the surface of the cerebral hemisphere (Casas-Torremocha et al., 2022) correlated with the section levels of the Paxinos and Franklin (2019) atlas. Functional subdivisions of areas M1 and M2 proposed by Tennant et al. (2011) and Zingg et al. (2014) were added onto this map. Axons labeled in the STN were likewise traced in 1:2 sections across this nucleus.

In the retrograde labeling experiments, we analyzed a full series of sections under 4-40X objectives. Labeled cell somata were counted (1:3 sections) across the entire brain and brainstem, separately for the sides ipsilateral or contralateral to the injection. To normalize between cases, labeled cells in each structure were compared as percentages over the total number of cells counted in each case. In addition, the spatial distribution of the labeled corticothalamic neurons was plotted on unfolded cortex maps.

### Axonal varicosity size measurement and comparison

Axonal varicosities are the predominant location of synapses in thalamic projection neurons (Rodriguez-Moreno et al., 2020), and differences in varicosity size correlate with the strength and dynamic properties of synapses (Rovó et al., 2012; Groh et al., 2014; Sherman, 2016). As a proxy for axonal varicosity size, we measured and compared maximal projection areas. Varicosities were identified as such when their diameter was at least twice that of the adjacent axonal segments. On live images on a Neurolucida platform under 100X oil-immersion optics, the varicosity contour was traced at its maximal focal plane, and its area measured using the Neurolucida software.

For the cortex, axon varicosities were sampled and compared among cytoarchitectonic area and layer subdivisions (Paxinos and Franklin, 2019) made visible by the tissue counterstain. Because cytoarchitectonic differences are not evident in the striatum, samples were compared among geometrically defined quadrants taken at three representative rostrocaudal levels (12 different sampling subregions in total). In every subregion containing labeled axons, at least 50 randomly selected varicosities were measured. Varicosities with projection areas close to the axon caliber (<0.2 μm^2^) were not included.

We used the two-tailed Mann–Whitney U test to compare value ranges and two-sample Kolmogorov–Smirnov test (K-S) to compare value distributions between structures. Statistical analysis was computed using GraphPad Prism 8 software. The threshold level of significance was set at *p < 0.05, **p < 0.01, and ***p < 0.001.

## Results

### Delineation of the mouse parafascicular nucleus

**Figure 1:**
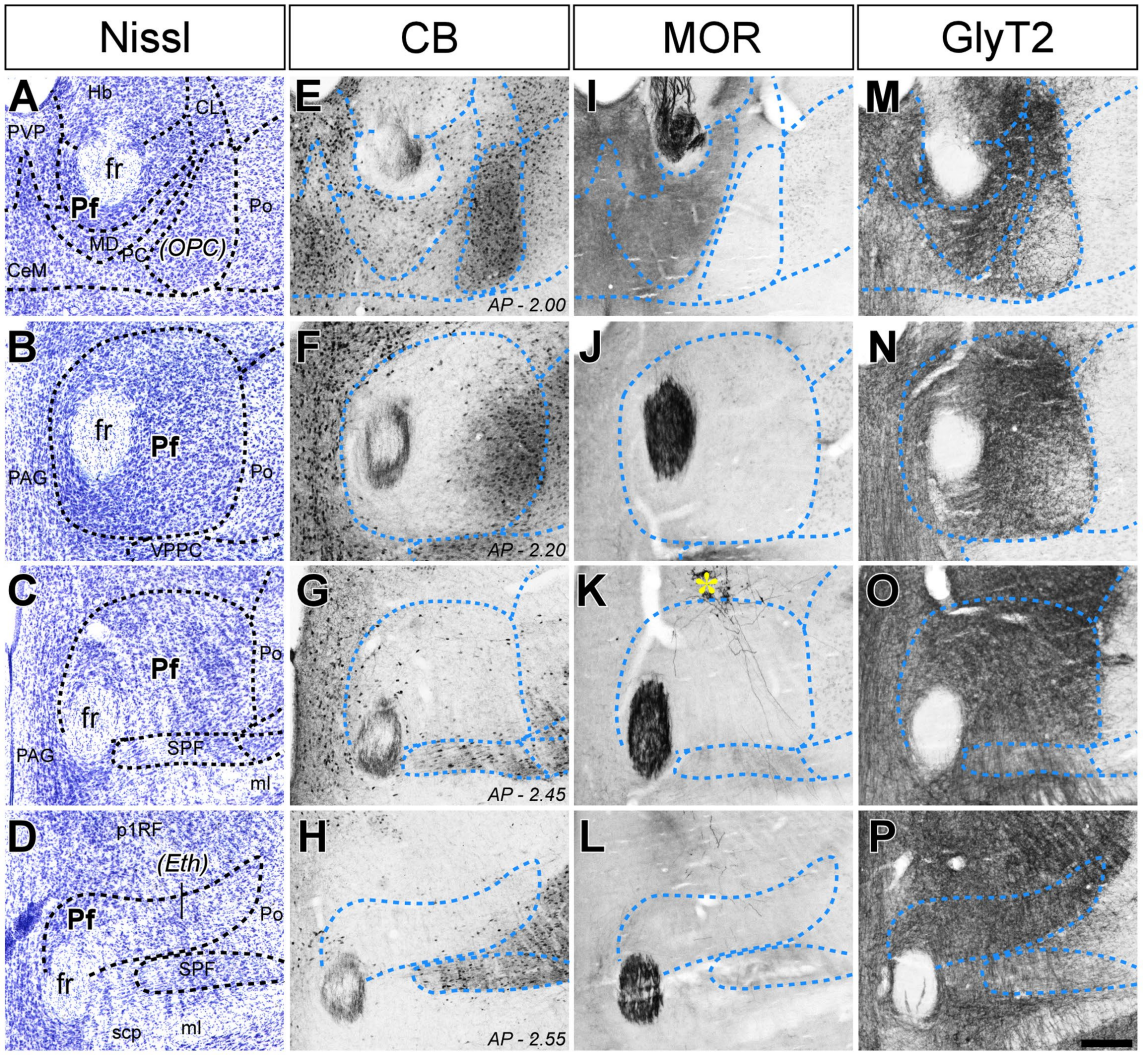
Cyto-and chemoarchitectonic heterogeneity of the mouse Pf in coronal sections. Parallel series of coronal sections stained with cresyl violet [Nissl; panels **(A–D)**], or immunolabeled for Calbindin 28 K [CB; panels **(E–H)**], μ-opioid receptor [MOR; panels **(I–L)**] or glycine transporter type 2 [GlyT2; **(M–P)**]. Coronal anterior–posterior (AP, in mm) levels for each row are indicated in panels **(E–H)**. In panel **(K)**, a yellow asterisk indicates some BDA-labeled pretectal cells in this experiment. Scale bar: 250 microns; CeM, central medial thalamic nucleus; CL, central lateral thalamic nucleus; Eth, ethmoid thalamic nucleus; fr, fasciculus retroflexus; Hb, habenula; MD, mediodorsal thalamic nucleus; ml, medial lemniscus; OPC, oval paracentral thalamic nucleus; p1RF, prosomere 1 reticular formation; PAG, periaqueductal gray; PC, paracentral thalamic nucleus; Pf, parafascicular thalamic nucleus; Po, posterior thalamic nucleus; PVP, paraventricular thalamic nucleus, posterior part; SPF, subparafascicular thalamic nucleus; VPPC, ventral posterior thalamic nucleus, parvocellular division.

**Figure 2:**
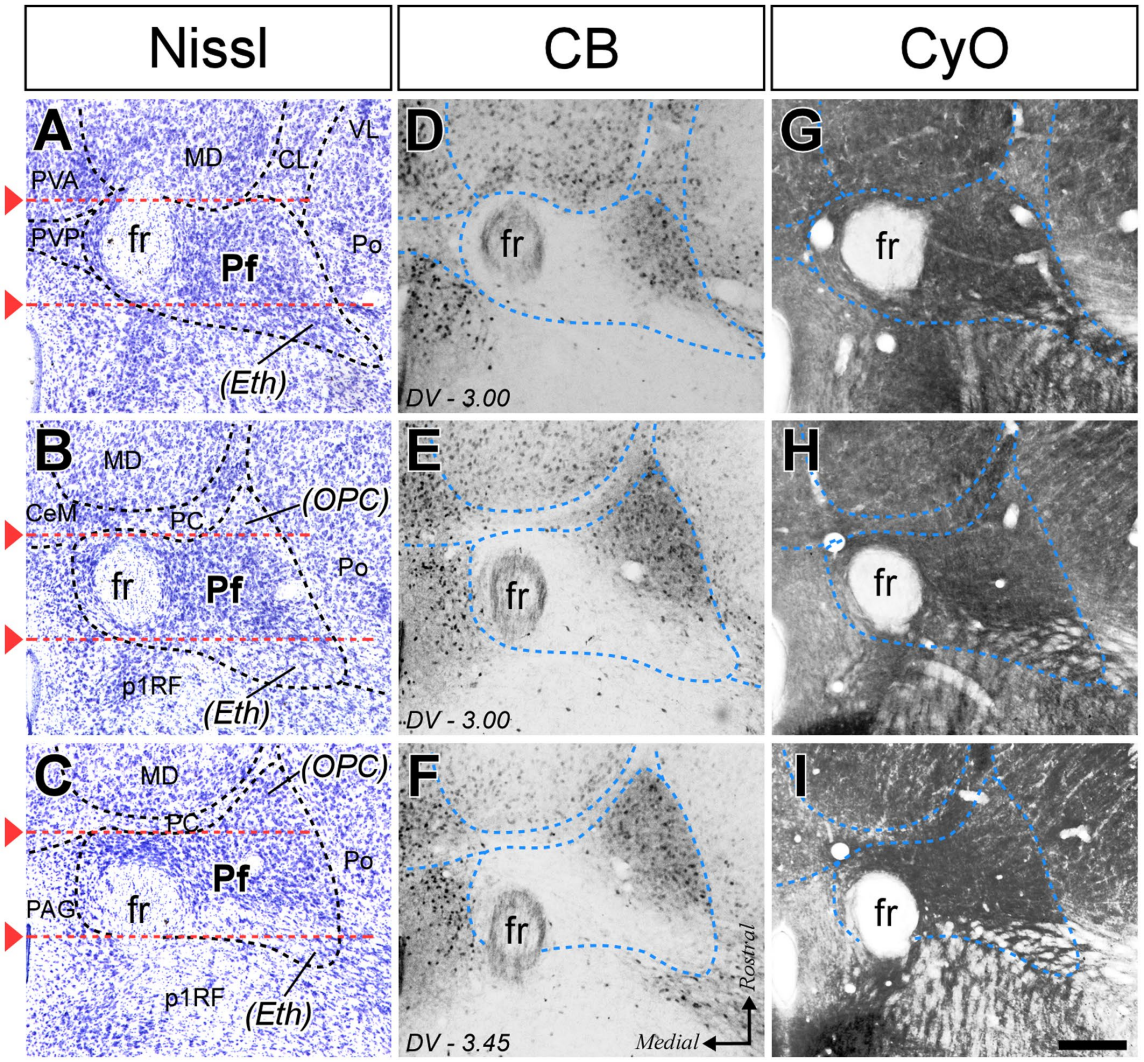
Cyto-and chemoarchitectonic heterogeneity of the mouse Pf in horizontal sections. Horizontal section images at various rostral (top) to caudal (bottom) levels, stained for Nissl **(A–C)**, CB **(D–F)**, and cytochrome C-oxidase histochemistry [CyO; **(G–I)**]. Red lines are used in **(A–C)** to indicate the coronal levels of the images shown in Figures 1A,D. Scale bar: 250 microns; PVA, paraventricular thalamic nucleus, anterior part; VL, ventral lateral thalamic nucleus. Other abbreviations as in Figure 1.

As a first step in the analysis of Pf input–output connection motifs, we delineated the Pf nucleus and examined its internal heterogeneity by comparing serial tissue sections made in different planes and subsequently stained for Nissl, CB, GlyT2, AChE, MOR, or CytOx (Figures 1, 2; Supplementary Figure S6).

In coronal sections, the nucleus appears as a circular mass of medium-sized cells that are more densely packed than the surrounding nuclei (Figure 1). In horizontal sections, the Pf is shaped as a parallelepiped (Figure 2). The nucleus is delineated from the laterally adjacent posterior and ventroposterior parvocellular thalamic nuclei by the internal medullary lamina (iml) fibers, which appear as a thin cell-free band in the Nissl-stained sections (Figures 1A–C, 2B,C), whereas the border of Pf is less distinct at rostromedial and dorsal levels; however, the heavy CB and MOR stainings in the paraventricular and anterior intralaminar thalamic nuclei provide a landmark for delineation (Figures 1E–I, 2D–F).

The anterolateral portion of Pf contains numerous CB-positive cell somata amidst a heavily immunopositive neuropil and shows relatively low GlyT2 staining (Figures 1E,F,M,N). In contrast, the caudal and medial portions of Pf show the opposite pattern (Figures 1E–H,M–P). Horizontal sections reveal that the CB-rich portion protrudes rostrally from Pf. On coronal sections, this portion may appear to be a relatively isolated cell group; for this reason, some previous studies identified it as a separate nucleus, the “oval paracentral nucleus” (Paxinos and Watson, 1986). Horizontal sections also show that the mediodorsal and paracentral thalamic nuclei bulge on the anterior border of Pf (Figure 2). As a result, coronal sections at about AP -2.00 mm show a medial fragment of Pf wrapped around the retroflex bundle (Figures 1A,E).

Caudally, the Pf dissolves among the pretecto-thalamic lamina fibers. In mice, this caudal Pf border is parallel to the coronal sectioning plane, and thus virtually undetectable in coronal sections (Figure 1D; Márquez-Legorreta et al., 2016). Previous studies based on coronal sections identified this region as a different (“ethmoid”) nucleus (Paxinos and Watson, 1986; Paxinos and Franklin, 2019). Horizontal sections show that the most caudal Pf cells simply intermingle with dorsoventrally-running fiber bundles (Figure 2).

### Anterograde labeling of Pf projection axons

To label and compare the output pathways originated in the various portions of Pf, we electroporated BDA into small compact neuron micropopulations. The analysis reported below is based on a total of 37 valid microdeposits (Supplementary Figure S1). Eight further deposits with tracer contamination along the pipette track or spreading into the adjacent thalamic nuclei were excluded. The valid deposits sampled most of the Pf volume, usually via more than one injection.

#### General pattern of labeling produced by BDA deposits in Pf

**Figure 3:**
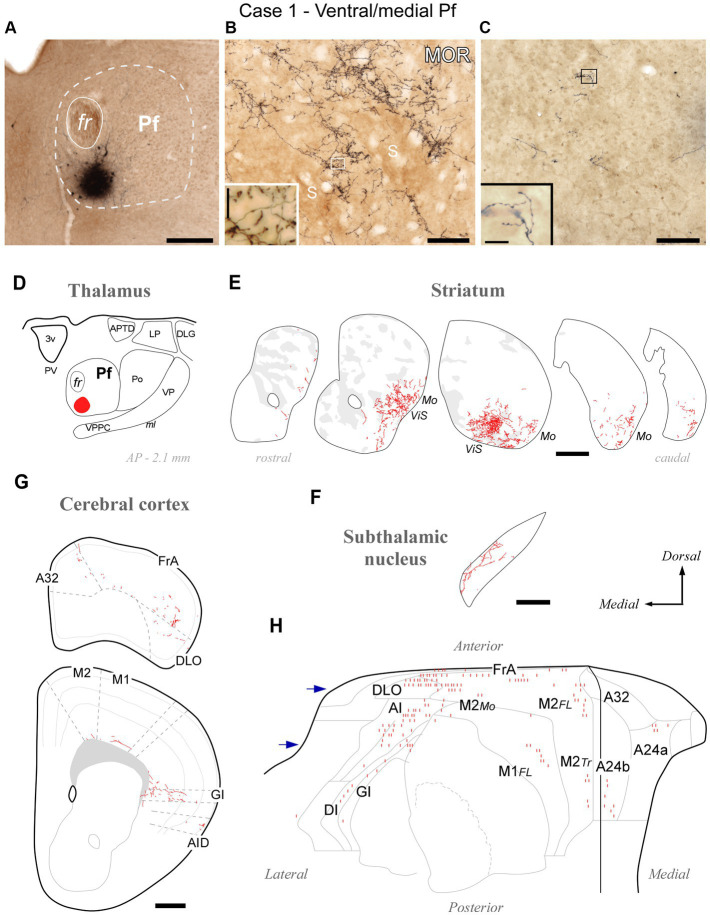
Divergent axonal projections to the striatum, subthalamic nucleus and cerebral cortex labeled by an injection in the ventral and medial domain of Pf (Case 1). **(A)** Center of the BDA deposit in experiment #1. **(B)** High-power image of thalamostriatal axonal arborizations. MOR immunohistochemistry counterstain. Striosomes (S) are visible as patches of slightly darker neuropil staining that are mostly avoided by the labeled axon terminals. **(C)** Labeled thalamocortical axon branches in the middle layers of area FrA. **(D)** Diagram illustrating the extent of the BDA deposit. **(E)** Coronal CPu sections profiles showing Neurolucida-drawn labeled thalamostriatal arborizations (in red). For reference, striosomes made visible by MOR immunolabeling in each section are also delineated (pale gray patches). For reference, functional territories of the mouse striatum where the axons labeled in this experiment are situated are indicated: Mo, mouth sensorimotor territory; ViS, visceral sensory territory. **(F)** Diagram of labeled Pf axon branches in STN. **(G)** Coronal section diagrams of the frontal cortex showing the labeled thalamocortical arborizations. **(H)** Serial plotting of the position and relative density of thalamocortical axons on an “unfolded” map of the anterior half of the cerebral cortex. In this diagram, axons are schematically represented by small line segments aligned along each section contour. For reference, the blue arrows indicate the levels corresponding to sections in panel **(G)**. Scale bars: 250 μm **(A,F)**; 100 μm **(B,C)**; 500 μm **(E,G)**. A32, cingulate cortex, area 32; A24a, cingulate cortex, area 24a; A24b, cingulate cortex, area 24b; AI, agranular insular cortex; AID, agranular insular cortex, dorsal part; APTD, anterior pretectal nucleus, dorsal part; DI, disgranular insular cortex; DLG, dorsal lateral geniculate nucleus; DLO, dorsolateral orbital cortex; FL, forelimb-related; FrA, frontal association cortex; GI, granular insular cortex; LP, lateral posterior thalamic nucleus; M1, primary motor cortex; M2, secondary motor cortex; Mo, mouth-related; PV, paraventricular thalamic nucleus; S, striosome; Tr, trunk-related; VP, ventral posterior thalamic nucleus. Other abbreviations as in Figure 1.

BDA-labeled Pf axons targeted the CPu, STN, and cerebral cortex. From the injection site, the axons extended in an anterolateral trajectory. Some traveled in the internal medullary lamina while other diverged across the posterior and ventral lateral nuclei of the thalamus. The axons crossed and left small collateral branches in a ventral zone of the thalamic reticular nucleus (TRN) between coronal bregma planes −0.60 and − 1.30. Beyond the TRN, they turned dorsally and extended along the internal capsule. The axons gave off some isolated thin branches to the globus pallidus (GP) and the entopeduncular nucleus (EPN); some of these branches ran caudally across the zona incerta to arborize in the STN and SNr. Other branches left the parent trunk in the striatal segment of the internal capsule and ramified in the striatum to form several densely branched terminal plexuses separated by label-free zones. Comparison with MOR immunolabeling revealed that these arborizations are always located in the matrix compartment of the striatum (Figure 3B). In all cases, thalamostriatal arborizations showed numerous varicosities and varicosity-tipped appendages were observed (Figure 3B, inset). An equivalent morphology has been reported in different mammals (Deschênes et al., 1996; Parent and Parent, 2005) and dubbed thalamostriatal “Type 2.” In addition, a few experiments labeled some arborizations with simpler elongated branches and *en passant* boutons (“Type 1”; Deschênes et al., 1996) that were mixed with the Type 2 terminals. The neurons that produced this type of terminal arborization were located ventrally and anteriorly in Pf.

The axons continued into the pallial white matter to finally reach the cerebral cortex. Here, they formed few and poorly branched varicose terminal arborizations scattered across all cortical layers but relatively more abundant in the infragranular layers (Supplementary Figure S2).

Over the common projection pattern just described, substantial differences were observed depending on the position of the BDA deposit within Pf.

#### Specific features of the labeling produced by injections in different portions of Pf

The BDA injections located in ventral/medial Pf (ventral to the fasciculus retroflexus, fr; cases #1 and #2, Supplementary Figure S1) selectively labeled axons in a ventral zone of the CPu, a region that receives its cortical input from orofacial-related sectors of areas M1, M2, Insular and S1 (Hintiryan et al., 2016). In addition, the BDA injections labeled axons in the ventral and medial portion of STN (Supplementary Figure S3), as well as in the dorsolateral orbital (DLO) and agranular insular (AI) areas of the cerebral cortex. A representative experiment (case #1) is illustrated in Figure 3.

**Figure 4:**
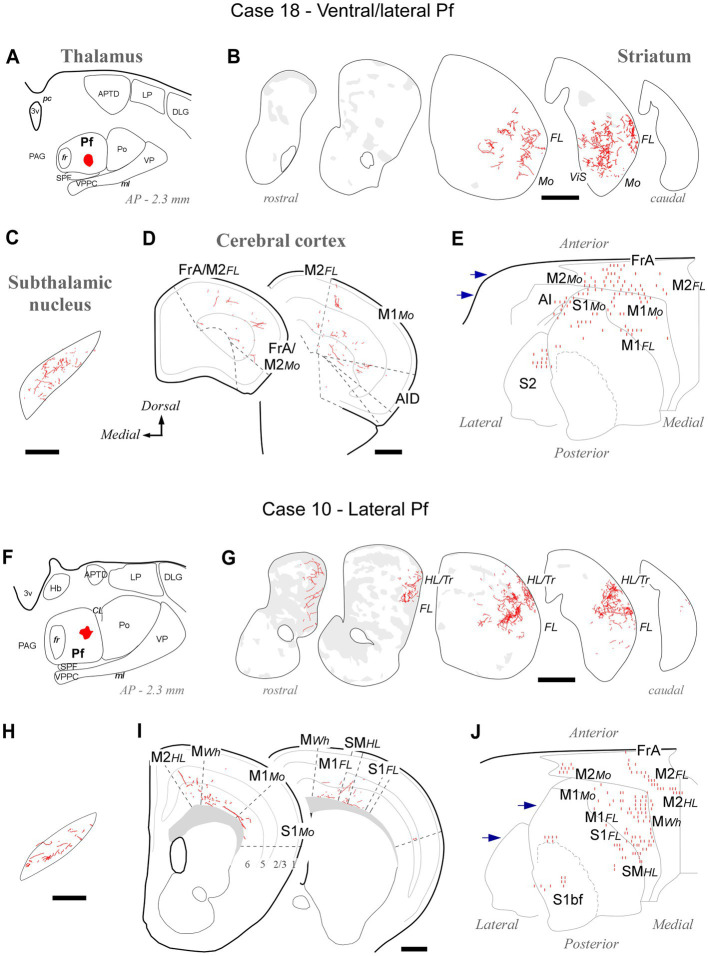
Labeling of axonal projections originated from either a ventral (Case 18, top) or an intermediate (Case 10, bottom) portion of the lateral Pf. Graphic conventions as in Figure 3. **(A,F)** Location of the BDA deposits. **(B,G)** Labeling in the striatum. **(C,H)** Labeling in the subthalamic nucleus. **(D,I)** Coronal section diagrams showing labeled thalamocortical arborizations. **(E,J)** Serial section plots of the cortical labeling. Scale bars: 250 μm **(C,H)**; 500 μm **(B,D,G,I)**. bf, barrel-field; HL, hindlimb-related; MWh, whisker-related motor cortex; S1, primary somatosensory cortex; SM, somatomotor region. Other abbreviations as in Figures 1, 3.

BDA deposits located ventrally and laterally in Pf (cases #18, #26, #27, #28, #29, #30, #31, #39, #41, #42) consistently labeled axonal arborizations in the ventral/lateral CPu. Case #18 is shown as representative (Figures 4A–E). These zones are associated with the processing of sensorimotor information from the inner and outer parts of the mouth (Hintiryan et al., 2016). In STN, axonal arborizations were labeled in the lateral two-thirds of this nucleus (Supplementary Figure S3). In the cortex, loose, poorly branched axons were labeled in sectors of M1, M2, S1, and AI reportedly associated with jaw, lips, or tongue movement/sensation (Tennant et al., 2011; Zingg et al., 2014; Hira et al., 2015).

Tracer deposits located laterally in Pf (cases #10, #12, #13, #19, #20, #24, #25; Supplementary Figure S1) invariably labeled dense terminal arborizations in a wide lateral CPu territory, a region associated with the processing of sensorimotor information from the forelimb (Carelli and West, 1991; Hintiryan et al., 2016). Case #10 is shown as representative of these experiments (Figures 4F–J). Labeled axons were also present in the dorsolateral two-thirds of STN (Supplementary Figure S3). In the cortex, sparse and poorly branched axons were visible in zones of M1 and M2 that have been associated with the control of forelimb movements (Tennant et al., 2011; Zingg et al., 2014). Moreover, most injections in lateral Pf labeled some axon branches in the motor vibrissal territory (M1/2 border, MWh, Tennant et al., 2011), as well as in the vibrissal region of S1 (S1bf; Figures 4I,J).

**Figure 5:**
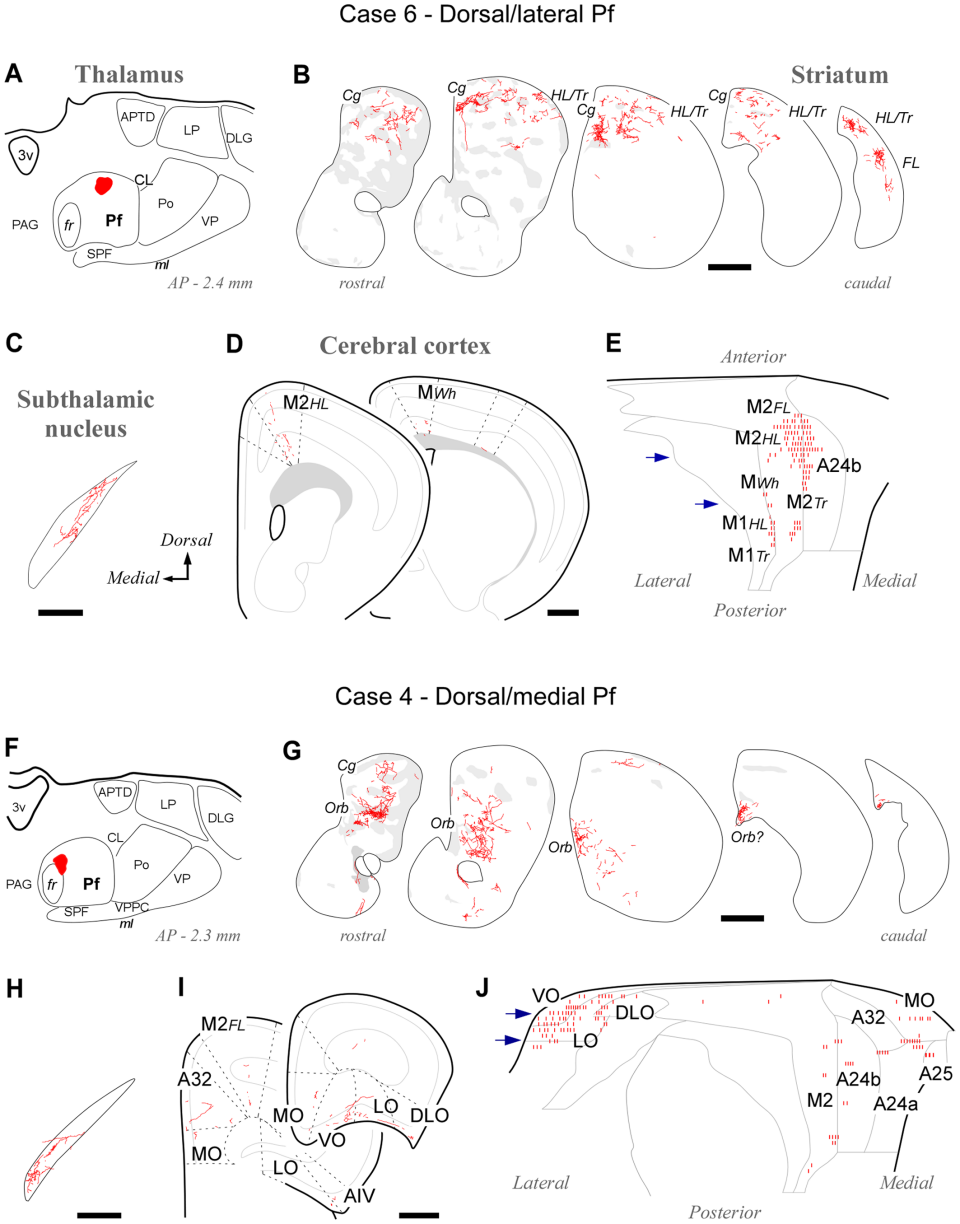
Labeling of axonal projections originated from either a dorsolateral (Case 6, top) or a dorsomedial (Case 4, bottom) portion of the Pf. Graphic conventions as in Figure 3. **(A,F)** Location of the BDA deposits. **(B,G)** Labeling in the striatum. **(C,H)** Labeling in the subthalamic nucleus. **(D,I)** Coronal section diagrams showing labeled thalamocortical arborizations. **(E,J)** Serial section plots of the cortical labeling. Scale bars: 250 μm **(C,H)**; 500 μm **(B,D,G,I)**. a25, cingulate cortex, area 25; AIV, agranular insular cortex, ventral part; Cg, cingulate cortex-related; LO, lateral orbital cortex; MO, medial orbital cortex; Orb, orbital cortex-related; Tr, trunk-related; VO, ventral orbital cortex. Other abbreviations, as in Figures 1, 3, 4.

Tracer deposits located dorsally and laterally in Pf at various anteroposterior levels (cases #5, #6, #7, #8, #21 and #22; Supplementary Figure S1) consistently labeled axonal arborizations in the dorsal third of the CPu along its entire rostrocaudal extent. This is a striatal region associated with the processing of sensorimotor information from the hindlimb and trunk (Hintiryan et al., 2016). In the cortex, the same experiments selectively labeled axons in M1 and M2 portions associated with the control of hindlimb and trunk movements (Tennant et al., 2011; Zingg et al., 2014). Case #6 is shown as representative (Figures 5A–E).

Tracer deposits located in the dorsal/medial Pf portions (cases #3 and #4; Supplementary Figure S1) labeled axonal arborizations mainly in an anteromedial CPu domain that receives its cortical input from cingulate, medial frontal and orbital cortical areas (Hintiryan et al., 2016). Labeled thalamocortical arborizations were located mainly in medial (A24a, A24b, and A32) and orbital frontal (MO, LO, and VO) areas. Case #4 is shown as representative (Figures 5F–J).

#### Labeling produced by injections at the rostral or caudal poles of Pf

As an additional criterion for determining the rostral and caudal borders of Pf, we found it informative to examine the connectivity patterns revealed by injections at or near these borders.

**Figure 6:**
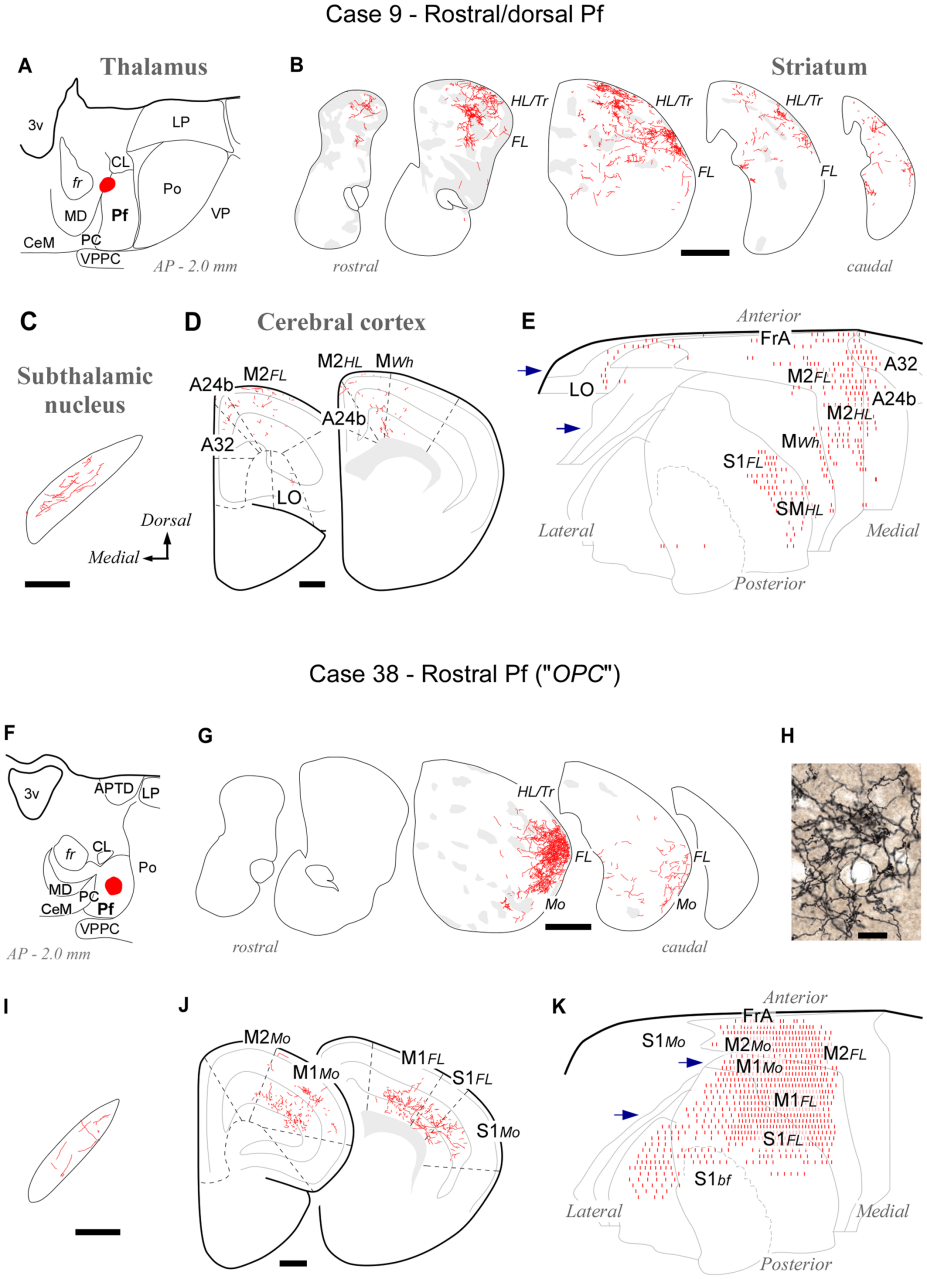
Labeling of axonal projections originated from either a dorsal (Case 9, top) or a ventral (Case 38, bottom) portion of the rostral Pf tip. Graphic conventions as in Figure 3. **(A,F)** Location of the BDA deposits. **(B,G)** Labeling in the striatum. **(H)** Microphotograph showing type II thalamostriatal axons labeled from the ventral part of the rostralmost Pf (“OPC”) arborizing in the striatum. **(C,I)** Labeling in the subthalamic nucleus. **(D,J)** Coronal section diagrams showing labeled thalamocortical arborizations. (E,K) Serial section plots of the cortical labeling. Scale bars: 250 μm **(C,I)**; 500 μm **(B,D,G,J)**; 100 μm **(H)**. Abbreviations as in Figures 1, 3–5.

At its rostral end, the Pf abuts the mediodorsal and paracentral thalamic nuclei (Figures 1, 2). A deposit in this portion, near the fr (AP -2.0; case #9, Figures 6A–E; Supplementary Figure S1), labeled numerous type 2 thalamostriatal arborizations in the dorsolateral portion of the CPu, which is a striatal region associated with the processing of sensory/motor information from the limbs and trunk (Carelli and West, 1991; Hintiryan et al., 2016). In turn, a Pf deposit situated in a rostral and ventral portion of the nucleus (the region labeled as a separate “OPC” nucleus by Paxinos and Franklin, 2019; case #38, Figures 6F–J; Supplementary Figure S1) labeled numerous type 2 thalamostriatal arborizations in the lateral portion of the CPu (forelimb and mouth-related regions; Carelli and West, 1991; Hintiryan et al., 2016). In addition, these two rostral cases labeled thalamocortical axons mainly in M1, M2 and S1. Case #9 injection labeled forelimb and hindlimb-related sectors of the above areas, whereas the injection in case #38 labeled sectors associated with orofacial and forelimb movement/somatosensation (Figure 6; Tennant et al., 2011; Zingg et al., 2014). It is of note that labeled thalamocortical arborizations were unusually profuse in case #38, probably because of some involvement of the paracentral intralaminar thalamic nucleus by this injection.

**Figure 7:**
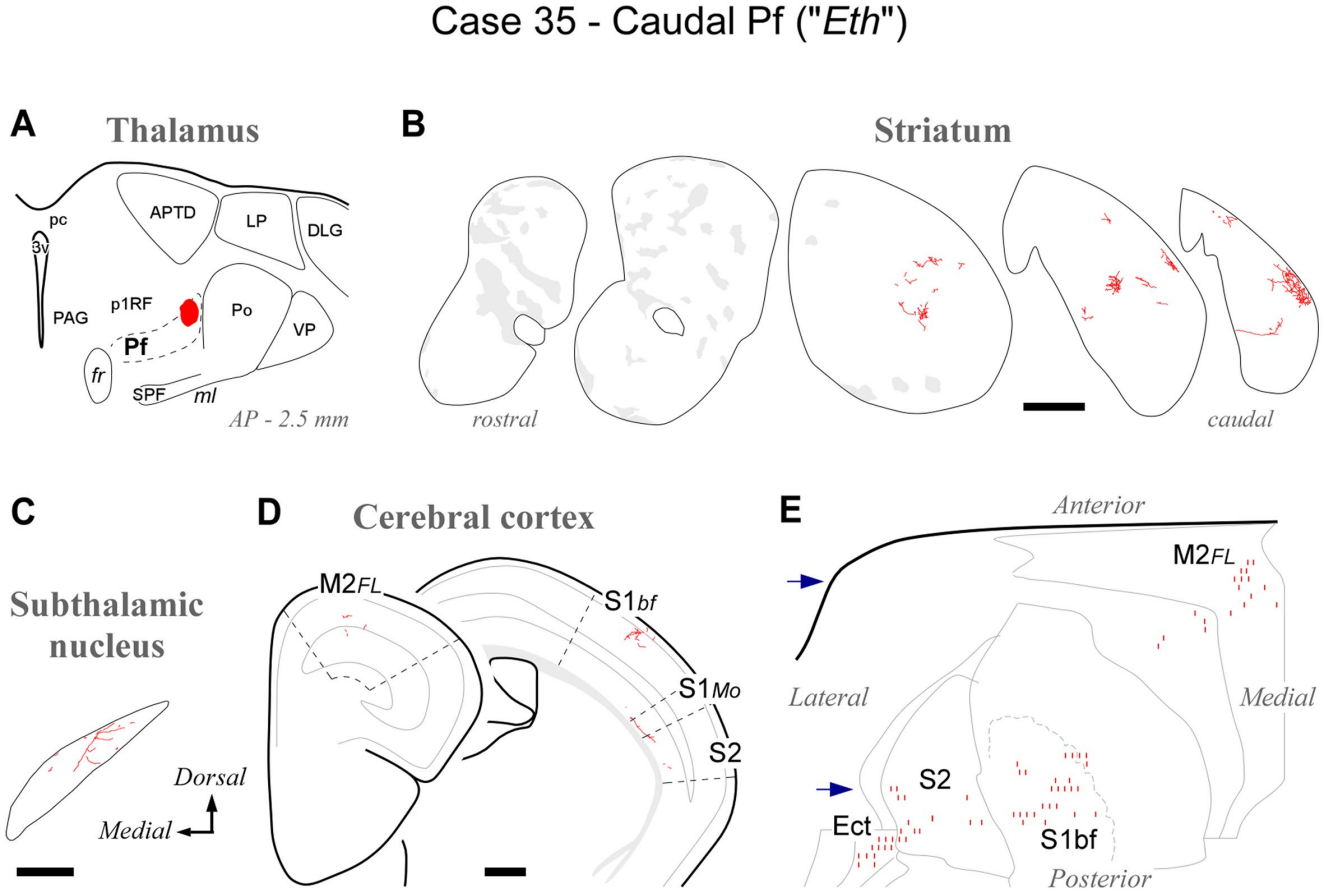
Labeling of axonal projections originated from the caudalmost cluster of Pf cells (“Eth nucleus”; case 35). Graphic conventions as in Figure 3. **(A)** Location of the BDA deposit. **(B)** Labeling in the striatum. **(C)** Labeling in the subthalamic nucleus. **(D)** Coronal section diagrams showing labeled thalamocortical arborizations. **(E)** Serial section plots of the cortical labeling. Scale bars: 250 μm **(C)**; 500 μm **(B,D)**. Ect: ectorhinal cortex. Other abbreviations, as in Figures 1, 3–6.

At its caudal end, the rodent Pf dissolves among the fibers of the pretectothalamic lamina (Figures 1, 2). BDA injections in this zone up to about AP -2.55 still labeled type 2 thalamostriatal axons, as well as some thalamocortical and thalamosubthalamic arborizations (case #35, Figure 7; Supplementary Figure S1).

Overall, the connection data support the notion that the caudal OPC nucleus, rostrally, and the Eth nucleus, caudally, may be better understood as extensions of Pf.

### Pf projections to STN

**Figure 8:**
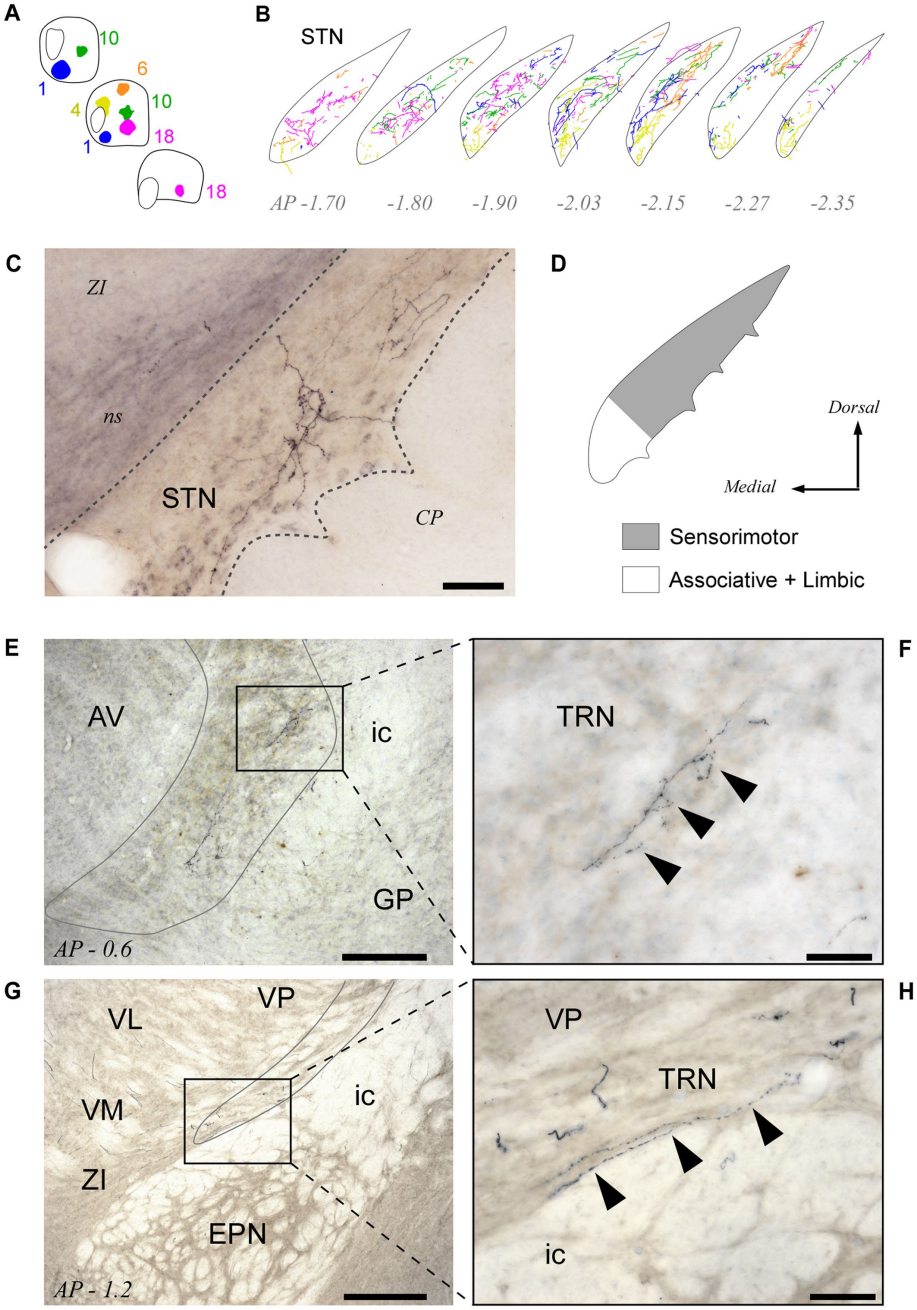
Pf projections to the subthalamic (STN) and thalamic reticular (TRN) nuclei. **(A,B)** Neurolucida^®^ drawings of the STN projection from the five BDA injection cases depicted in Figures 3-5 (Panel “**A**”) are overlaid in panel “**B**” to illustrate the fine spatial organization of this projection. (C) Microphotograph of a labeled thalamosubthalamic axon arborization (Case 6; see also Figure 5). **(D)** Summary diagram of the Pf-STN projection. **(E–H)** Microphotographs taken from two different BDA experiments at 10x **(E,G)** and 40x **(F,H)** magnification show that axons from Pf neurons leave a few varicose branches upon crossing through the TRN on their way to the striatum and cortex. Scale bars: 250 μm **(E,G)** or 50 μm **(C,F,H)**. AV, anteroventral thalamic nucleus; CP, cerebral peduncle; EPN, entopeduncular nucleus; GP, globus pallidus; ic, internal capsule; ns, nigrostriatal bundle; STN, subthalamic nucleus; TRN, thalamic reticular nucleus; VM, ventral medial thalamic nucleus; ZI, zona incerta. Other abbreviations, as in previous figures.

All BDA deposits in Pf, including its rostral or caudal pole zones, labeled axon arborizations in the STN. Their distribution within this nucleus varied according to the position of the injection site in the Pf (Figures 8A,B; see also Supplementary Figure S3). Axons from the lateral portions of Pf innervated mostly the dorsolateral two thirds of the STN, whereas axons from the medial Pf targeted the medialmost zone of the STN (Figures 8A–D).

### Analysis of Pf axon varicosity sizes in the striatum, cortex and subthalamic nucleus

**Figure 9:**
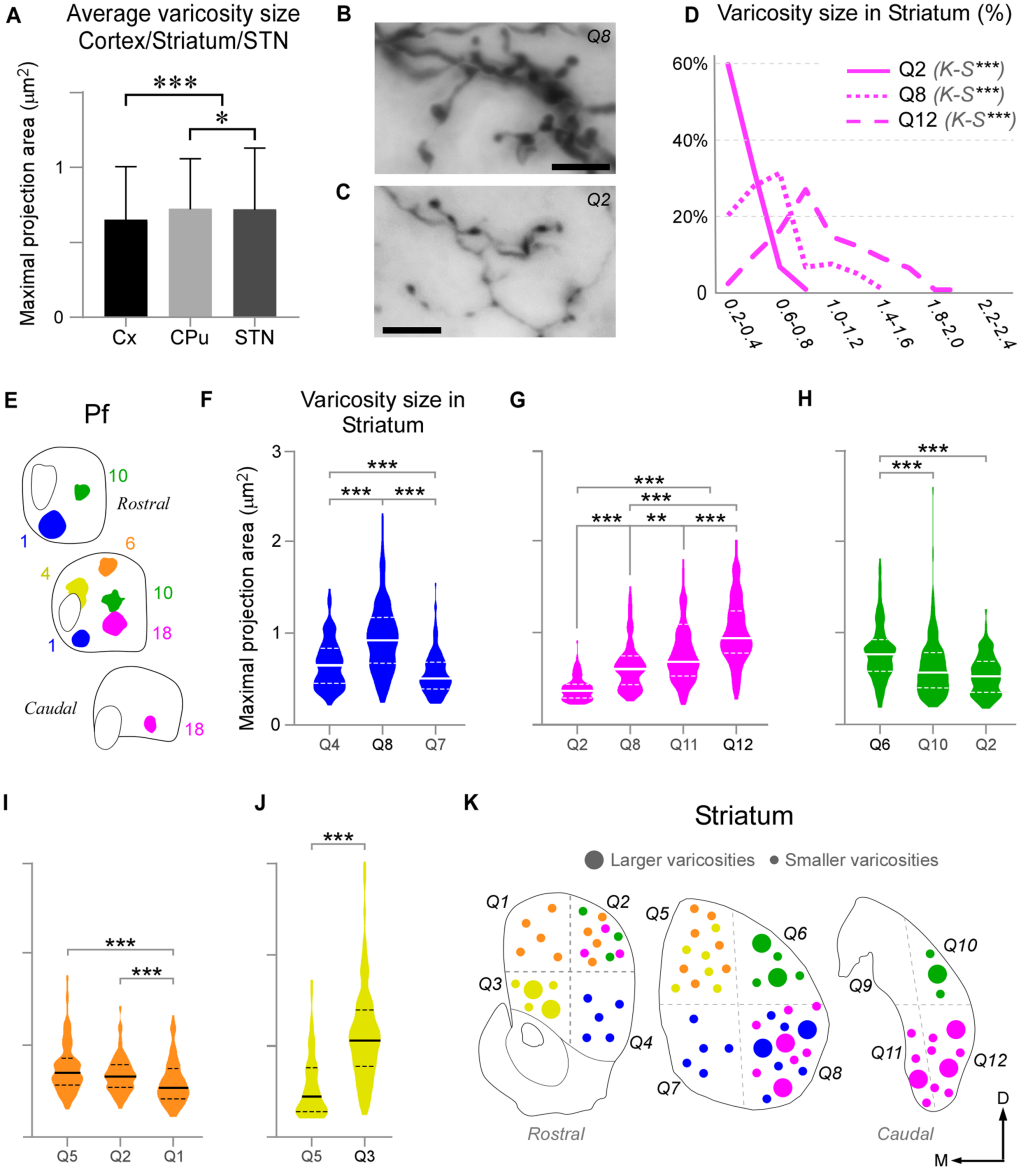
Pf axon varicosities show region-specific size differences. **(A)** Comparison of median sizes (maximal projection area, in μm^2^) of axon varicosities labeled in the cerebral cortex (Cx), striatum (CPu), or STN by Pf injections. Results from six cases are averaged in this chart. **(B–D)** Comparison of the sizes of the thalamostriatal axon varicosities labeled in different geometrical quadrants (“Q”) and anterior–posterior levels of the striatum by a BDA injection in the ventral/lateral portion of Pf (Case 18). Panels B and C show high-magnification images of the morphology of thalamostriatal axon arborizations containing large and small **(B)** and only small **(C)** varicosities. Scale bars: 10 μm. Panel **(D)** shows the quantification of size (maximal projection area) range distributions of the axon varicosities in different quadrants. Kolmogorov-Smirnoff (K-S) paired comparisons. Levels of significance are indicated by asterisks: *p < 0.05, **p < 0.01, ***p < 0.001. **(E–J)** Axon varicosity size distributions and paired comparisons across different quadrants of the striatum in five Pf injection experiments **(E)**. In each plot, a continuous line represents the median, while dashed lines represent interquartile ranges. Mann–Whitney U test (MW) paired comparisons. Levels of significance are indicated by asterisks: *p < 0.05, **p < 0.01, ***p < 0.001. The case in **(G)** is the one illustrated also in panels **(B–D)**. **(K)** Cartoon diagram summarizing the statistical analyzes in panels **(F–J)**. Three coronal section levels of the striatum are shown, and crossed dashed lines are used to separate the “quadrants” among which varicosities were compared. Large or small circles represent larger or smaller varicosity populations. Colors as in panel **(E)**.

We measured and compared the size of axon varicosities in the CPu, cortex and STN labeled by the Pf injections. Median values indicate that, in general, CPu and STN are innervated by slightly larger axon varicosities than cortex (0.6590 μm^2^, 0.6013 μm^2^ and 0.5451 μm^2^, respectively; Figure 9A). Although small, these differences are statistically significant (two-sample Mann–Whitney test, M-W; *p* < 0.05).

As described earlier, the heavy thalamostriatal Pf projections labeled in each of our BDA experiments focused mainly in a relatively precise functional sector of the striatum (Carelli and West, 1991; Hintiryan et al., 2016). However, some axonal arborizations spread to adjacent striatal sectors. We compared the size of the thalamostriatal varicosities across the various striatal sectors containing labeled axons in a given experiment (Figures 9B–K). For this comparison, we divided the CPu in four quadrants at three anteroposterior levels and measured their labeled varicosity sizes (see Methods section; Figure 9K). The analysis revealed that, as a general rule, the striatal zone/s more densely innervated by a given Pf portion contains varicosities that are slightly, but significantly, larger than those in other CPu sectors of the same experiment (two-tailed Kolmogorov–Smirnov test, K-S; *p* < 0.01). These differences were always present, although most clear in the experiments with an injection located in ventral and/or medial Pf sectors (Figures 9F,G,J).

### Retrograde labeling of cortical and subcortical inputs to Pf

**Figure 10:**
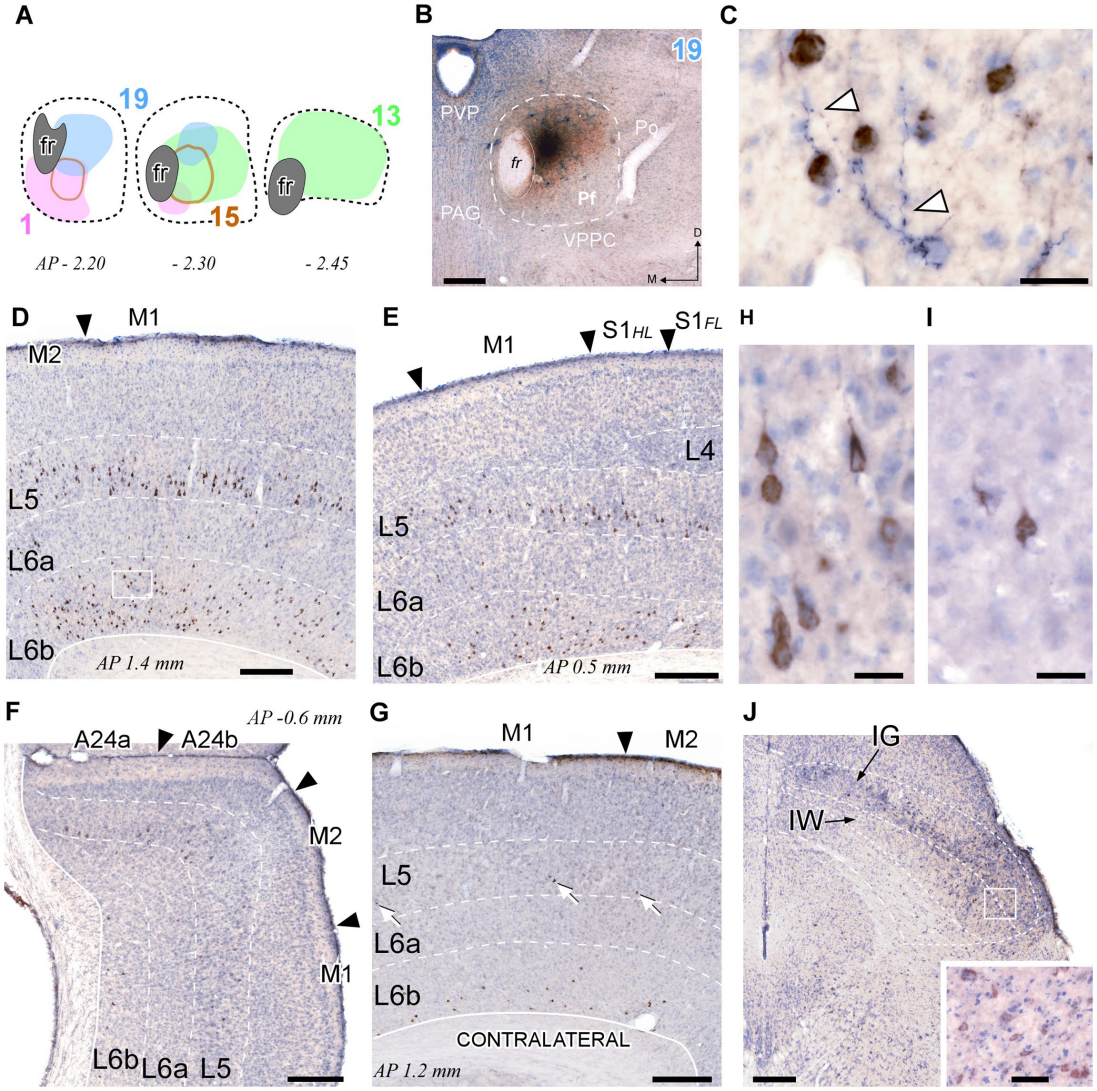
Retrograde CTb labeling of corticothalamic and superior colliculus neurons projecting to Pf. **(A)** Diagram showing the extent of four different BDA + CTb injection cases (Supplementary Figure S1). **(B)** The injection site in Case 19 **(B)** is shown as a representative example. The CTb deposit is visible as a brown DAB precipitate, whereas the BDA deposit appears as a black DAB-nickel core. Thionin counterstain. **(C)** High-magnification detail of the neuronal somata labeled by CTb transport (in brown) in L6 of the motor cortex [area M1, inset in panel **(D)**]. An isolated thalamocortical axon branch (arrowheads) labeled by the anterograde transport of BDA (blue-black) can be seen among the corticothalamic cell somata. **(D–F)** Coronal sections samples illustrating the labeling in the cerebral cortex ipsilateral to the injection. Large numbers of CTb-labeled corticothalamic cell bodies are visible in the lower part of L5 (putative L5b), and in L6b. The scant, isolated thalamocortical axons labeled by BDA are barely visible at this magnification [compare with panel **(C)**]. **(G)** Corticothalamic cell bodies labeled in the hemisphere contralateral to the injection. White arrows indicate corticothalamic L5 cells. Labeled somata are also visible in L6b. **(H,I)** High-magnification detail of labeled L5 pyramidal cells in area M1 of the injected **(H)** or contralateral **(I)** hemisphere. **(J)** Retrogradely labeled neurons in the intermediate gray (IG) and white (IW) layers of the superior colliculus. Inset: High magnification detail of labeled cells in the intermediate layers. Scale bars: 250 μm **(B,D,E,F,G)**; 25 μm **(C,H,I)**; 50 μm [inset in **(J)**].

Because inputs define the signal computations that thalamic cells may carry (Jones, 2007; Acsády, 2022), we set out to elucidate the sources of input to each portion of Pf and to correlate them with the projection arising from the same Pf portions. To this end, we made iontophoretic injections of a mixture of BDA + CTb in Pf. Four valid cases were centered in different Pf zones and did not spread beyond the nucleus boundaries (Figures 10A,B). In these experiments, we counted the retrogradely labeled cell somata on both sides of the midline in one-sixth sections throughout the brain and brainstem.

**Figure 11:**
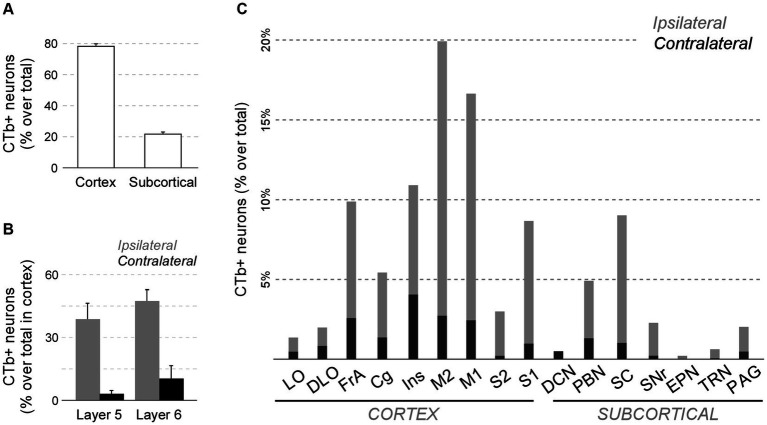
Quantitative analysis of neurons projecting to Pf from cortical and subcortical areas of the brain and brainstem. **(A)** Average percent of retrogradely labeled neurons in cortical or subcortical structures over the total. **(B)** Average percent of retrogradely labeled L5 or L6 cells for four sensorimotor cortical areas (M1, M2, S1, S2). Ipsilateral (gray) and contralateral (black) labeling data are represented separately. **(C)** Percentage of retrogradely labeled cell bodies in different cortical or subcortical structures is indicated. Ipsilateral or contralateral data are separately indicated. To normalize between experiments (*n* = 4), the percent of cells labeled in each structure over the total of cells counted in the experiment is averaged. For simplicity, we grouped under “Cg” the various areas in the medial frontal cortex (A24, A25, A32). Individual case data are available as Supplementary Figure S4.

This analysis revealed that the cerebral cortex is, by far, the main source of inputs to Pf (~78% of total counted CTb-labeled cells; Figure 11A). On average, the areas that originated the most robust corticothalamic projections were M2 (19.7%), M1 (16.5%), Ins (10.8%), FrA (9.7%), S1 (8.6%) and Cg (5.3%; Figure 11C). A large fraction (~45%) of the corticothalamic cells were labeled in the lower tier of L5 (putative layer 5b). A Numerous cells were also labeled in L6b, along with a few cells in L6a (Figures 10D–G, 11B). Although most of the labeled corticothalamic neurons were in the same side of the injected Pf, a small but consistent number of cells (~15% of the total) was labeled in the opposite hemisphere. These contralateral neurons were labeled in L6b; remarkably, however, a contingent of L5b cells was consistently labeled as well (Figures 10G,I; Supplementary Figure S4).

**Figure 12:**
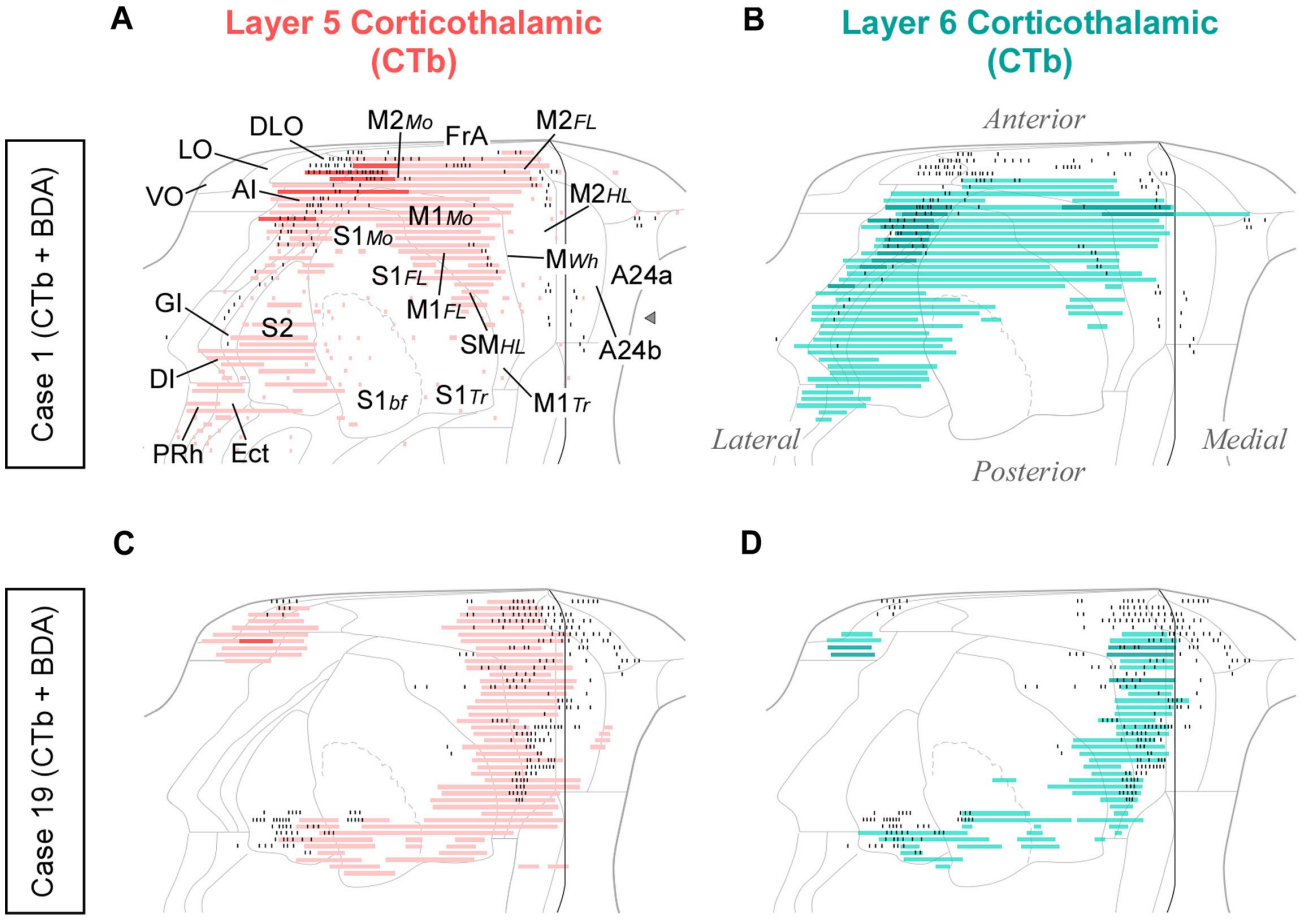
Distribution of labeled corticothalamic layer 5 (red) or layer 6 (turquouise) neurons following CTb injections in Pf. **(A,B)** Cells labeled by a CTb injection in ventral/medial Pf (Case 1, Figure 10); **(C,D)** Cells labeled by an injection in dorsal Pf (Case 19, Figure 10). Labeling is displayed on unfolded cortex maps. Due to their high density, labeled cells are represented as continuous bars. Higher saturation indicates zones of higher cell density. In the same experiments, BDA was co-injected with CTb, thus producing anterograde labeling of Pf axons; for comparison, the position of these axons is shown as black dots. PRh: perirhinal cortex. Other abbreviations, as in previous figures.

In each of these double-labeling experiments, the ipsilateral areas containing the largest populations of corticothalamic cells roughly matched the distribution of the labeled thalamocortical Pf axons (Figure 12). The BDA-labeled axons were readily distinguished by their morphology and color of staining (see Methods section). The massive abundance of CTb-labeled cells contrasted starkly with the scarcity of BDA labeled thalamocortical axons, hence providing a vivid image of the highly unequal weight of the corticothalamic vs. thalamocortical Pf connections (Figures 10C–G; Supplementary Figure S2).

In addition to cortex, numerous cells (~22% of the total; Figures 11A,C) were labeled in subcortical structures. Among these, the main contingent was localized in the intermediate and deep laminae of the superior colliculus (SC; 9.4%; Figure 11C). Regardless of the position of the CTb deposit within Pf, these cells were predominantly located in lateral portions of the intermediate layers (Figure 10J).

In addition, smaller neuron populations were labeled in the parabrachial nuclei (PBN; ~5.0%), the SNr (2.2%), the periaqueductal gray (2.0%), the TRN (0.6%), and the contralateral deep cerebellar lateral nuclei (0.5%; Figure 11C). Smaller contingents of cells were labeled in the mesencephalic reticular formation, with additional cells labeled in the oral pontine reticular formation, in the ventral zona incerta, and the laterodorsal tegmental and pedunculopontine nuclei.

While retrograde labeling patterns were overall consistent between cases, the abundance of labeled cells in each structure varied with the position within Pf of each tracer deposit (Supplementary Figure S4). For example, a CTb deposit situated ventrally and medially within Pf (case #1) labeled corticothalamic cells mainly in AI, DLO, and the adjacent lateral zones of FrA/M2 (Figures 12A,B). A deposit situated more laterally in Pf (case #15) labeled corticothalamic cells chiefly in M1 (Supplementary Figure S4). In contrast, deposits that involved more dorsal portions of Pf (cases #13 and #19) labeled corticothalamic cells mostly in rostral M2 (Figures 12C,D; Supplementary Figure S4).

### Anterograde labeling of cortical and subcortical inputs to Pf

To gain further insight into the distribution of the various input systems within Pf, we examined the anterograde axonal labeling produced by adenoassociated GFP vectors in the structures identified as main sources of cortical and subcortical input in the CTb injection experiments (AAV-GFP; Allen Institute for Brain Science; Oh et al., 2014).

**Figure 13:**
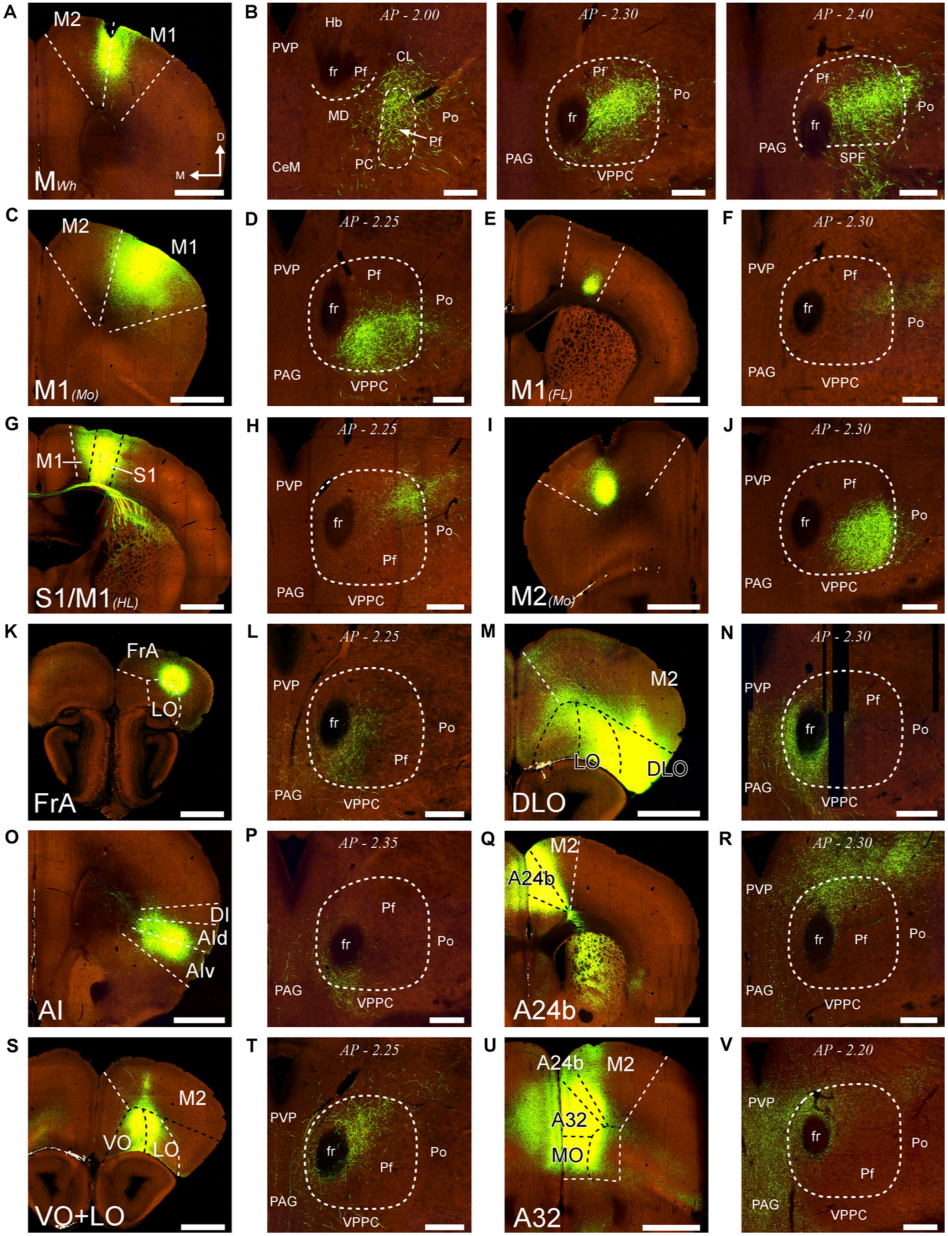
Distribution within Pf of corticothalamic axon terminals from different cortical areas. Two-photon tomography image samples from experiments in which AAV vectors able to drive the expression of high levels of fluorescent protein were injected in different areas. **(A,B)**: Corticothalamic projection labeled by an AAV injection in M2 and M1 (putative Wh zone). Three sections throughout the entire rostrocaudal extent of the Pf are shown, as a representative example of our analysis methodology **(C,D)**: Projection from M1 (putative mouth zone) **(E,F)**: Projection from M1 (putative forelimb zone). **(G,H)**: Projection from M1/S1 (putative hindlimb zone). **(I,J)**: Projection from M2 (putative Mo zone). **(K,L)** Projection from FrA. **(M,N)** Projection from DLO. **(O,P)** Projection from AI. **(Q,R)** Projection from area 24b of the cingulate cortex. **(S,T)** Projection from VO and LO. **(U,V)** Projection from A32/MO areas. Images from the Allen Institute Mouse Connectivity Projection datasets https://connectivity.brain-map.org/. Experiment IDs: 183617432 **(A,B)**; 584,903,636 **(C,D)**; 159,651,060 **(E,F)**; 100,141,273 **(G,H)**; 552,757,477 **(I,J)**; 293,433,996 **(K,L)**; 180,709,230 **(M,N)**; 262,536,037 **(O,P)**; 496,576,666 **(Q,R)**; 183,618,845 **(S,T)**; 478,376,197 **(U,V)**. AP: bregma level in mm. Scale bars: 1000 μm **(A,C,E,G,I,K,M,O,Q,S,U)**; 250 μm **(B,D,F,H,J,L,N,P,R,T,V)**. Abbreviations, as in previous figures.

First, we analyzed AAV-GFP injections in cortical areas M2, M1, S1, DLO, VO, FrA, LO, AI, and cingulate (Figure 13). Despite the relatively large size of the injections, each labeled a dense plexus of terminal arborizations limited to a restricted portion of Pf. The position of the plexuses varied among experiments along the dorsal and ventral axes of Pf but not along its rostrocaudal axis. In addition, the experiments detected a consistent contralateral corticothalamic projection, which was most robust from DLO (Supplementary Figure S5).

**Figure 14:**
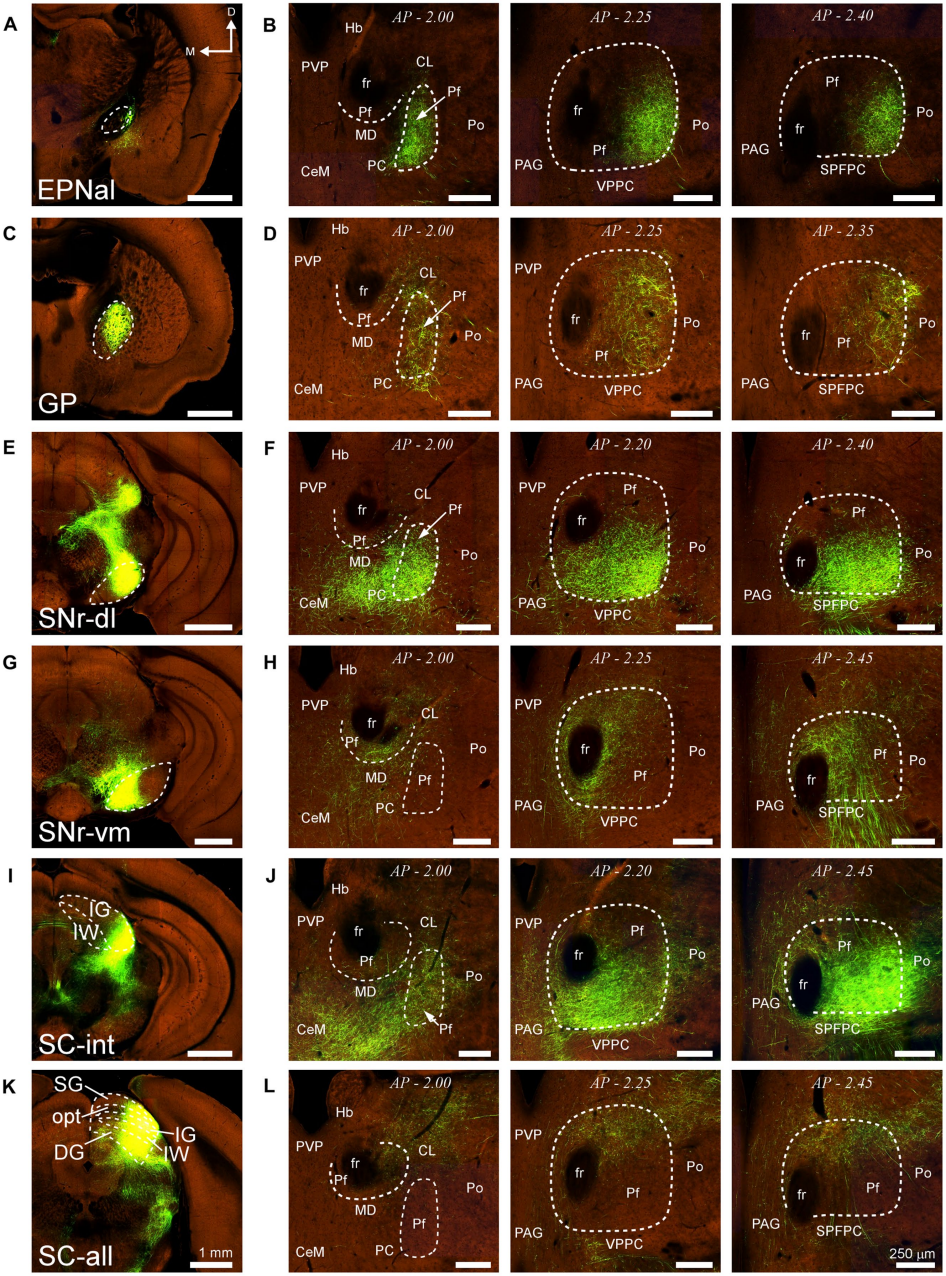
Projections to Pf from different subcortical regions. Two-photon tomography image samples from experiments in which AAV vectors able to drive the expression of high levels of fluorescent protein were injected in some of the subcortical structures that target Pf. **(A,B)** Projection from the entopeduncular nucleus, anterolateral part. **(C,D)** Projection from the globus pallidus. **(E–H)** Projection from the substantia nigra pars reticulata. Injections in either a dorsolateral **(E,F)** or a ventromedial **(G,H)** region are shown. **(I–L)** Projection from the superior colliculus. Injections in either a lateral **(I,J)** or a central-lateral **(K,L)** region are shown Images from the Allen Institute Mouse Connectivity Projection datasets https://connectivity.brain-map.org/; experiment IDs: 539498984 **(A,B)**, 511,942,270 **(C,D)**, 478,096,249 **(E,F)**, 158,914,182 **(G,H)**, 175,158,132 **(I,J)**, 128,001,349 **(K,L)**. AP: bregma level in mm. Scale bars: 1000 μm **(A,C,E,G,I)**; 250 μm **(B,D,F,H,J)**. DG, deep gray layer; opt, optic tract layer; SC, superior colliculus; SC-all, superior colliculus, all layers; SC-int, superior colliculus, intermediate layers; SG, superficial gray layer; SPFPC, subparafascicular thalamic nucleus, parvicelular part. Rest as in previous figures.

Similarly, AAV-GFP injections in subcortical structures such as the output nuclei of the basal ganglia system (EPN and SNr) or the PBN (not shown) labeled terminal axon plexuses in specific portions along the dorsal and ventral axes of Pf (Figures 14A–H). A rough but evident mediolateral topography existed also in the projection from the SC (Figures 14I–L).

## Discussion

Our results show that the mouse Pf is a high-centrality hub of the motor system. It receives massive and partly bilateral input from frontal cortex lower L5 (putative L5b) and from the superior colliculus intermediate and deep layers. In turn, Pf neurons heavily innervate in multifocal yet orderly fashion the matrix compartment in all regions of the dorsal striatum (CPu). Additionally, the Pf neurons innervate the subthalamic nucleus and the cerebral cortex. The Pf thalamocortical axon arborizations are remarkably sparse and poorly branched, and do not form focal arborizations.

In the following sections, we first briefly discuss the delineation of the mouse Pf from adjacent cellular groups. Subsequently, we examine the input–output logic of the observed connectivity and terminal axon morphology. Finally, we compare the picture of the mouse Pf that emerges from the present dataset with the organization of the anthropoid primate CM-Pf.

### Delineation of the mouse Pf

In coronal sections, the lateral and ventral borders of Pf are sharply delineated by the internal medullary lamina. The medial border is less evident in Nissl-stained sections but detectable by the absence of calbindin-immunostaining, in contrast with the adjacent paraventricular and subparafascicular thalamic nuclei (Figure 1).

The rostral and caudal poles of the mouse Pf are difficult to establish by examining only coronal section images. Comparison with horizontal sections supports the notion that the “*oval paracentral nucleus*” (Paxinos and Watson, 1986) is a rostral protrusion of the Pf cell mass (Figure 2). Additional evidence for considering this cell group a rostral part of Pf derives from the analysis of thalamostriatal arborizations originating from this region. Unlike axons from other thalamic nuclei, which form extended arborizations consisting of relatively simple, elongated, and varicose branches (“Type 1” thalamostriatal axons), the Pf neuron axons typically give off multiple separate and richly branched terminal arborizations, with numerous short varicosity-tipped appendages (“Type 2” thalamostriatal axons; Deschênes et al., 1995). In our experiments, BDA injections in the “oval” rostral Pf labeled abundant Type 2 axonal arborizations (Figure 6H).

At its caudal end, the Pf neurons become progressively scattered among the myelinated bundles of the pretecto-thalamic fiber lamina (Márquez-Legorreta et al., 2016). The bundles pierce the Pf gray matter, hence the name “*ethmoid*” (“perforated”) coined for this region by Paxinos and Watson (1986). The morphology and connectivity of cells in this zone are indistinguishable from those of Pf cells (Deschênes et al., 1995). Our injections in this region labeled numerous Type 2 thalamostriatal axon arborizations, supporting the possibility that this region is a caudal part of Pf.

### Corticothalamic layer 5 and layer 6 inputs

Consistent with fragmentary observations in previous rat (Cornwall and Phillipson, 1988; Groenewegen and Berendse, 1994) and mice studies (Mandelbaum et al., 2019; Foster et al., 2021), our retrograde and anterograde data show that the mouse Pf receives its anatomically most robust input from the frontoparietal (M1, M2, FrA, S1), anterior limbic, and insular cortices. Occipital and temporal areas of the cortex do not innervate Pf.

**Figure 15:**
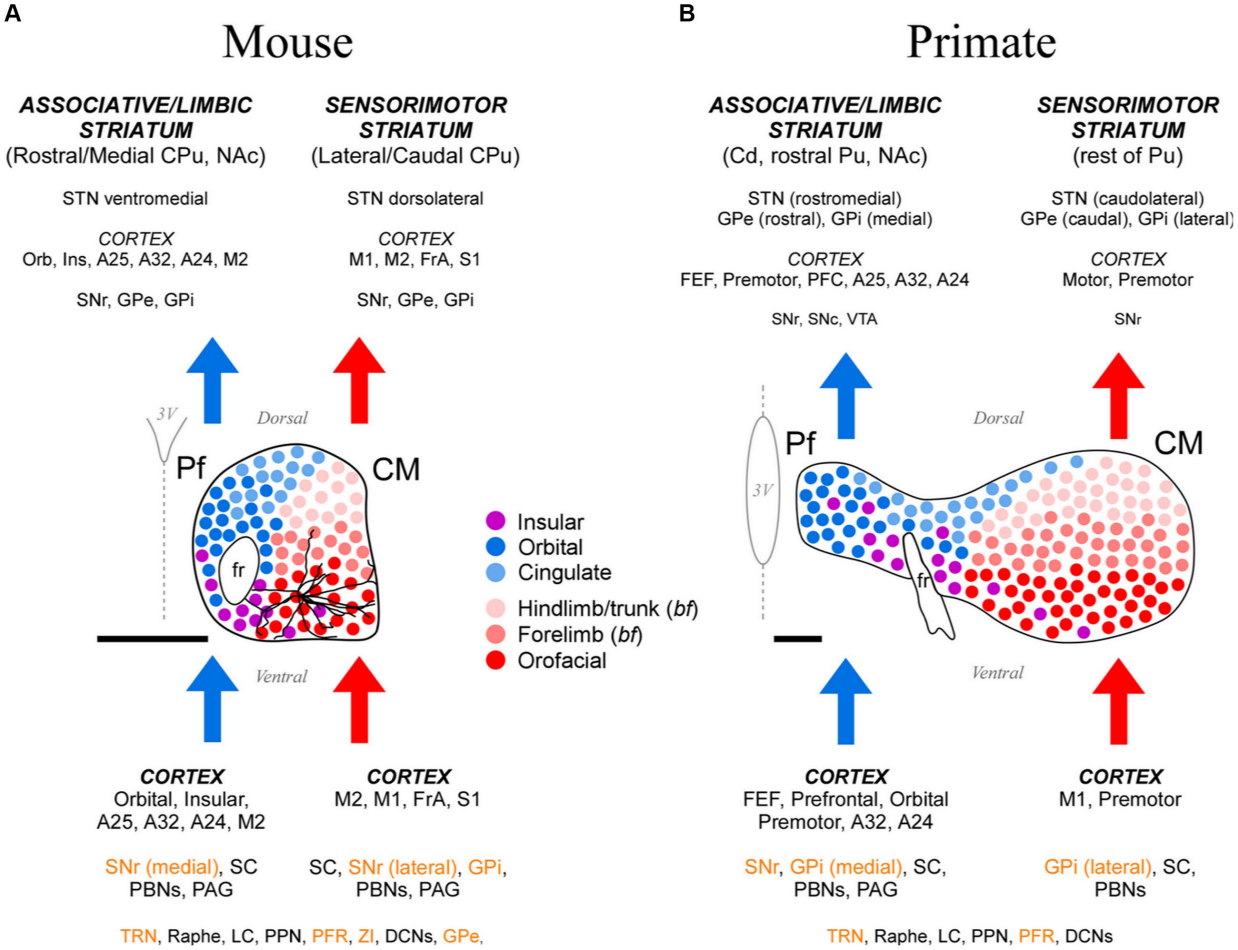
Cartoon comparison between the anatomical organization patterns of the mouse and the anthropoid primate CM-Pf. **(A)** Schematic representation of the mouse CM-Pf complex and its afferent (bottom) and efferent (top) connections as revealed by our analysis. Connections whose neurotransmission is known to be fundamentally inhibitory are shown in orange letters. For scale comparison, the somatodendritic morphology of a typical Pf neuron (taken from the Janelia Research Campus Mouselight database; #AA1439; Winnubst et al., 2019) is illustrated. Note that the long dendrites from an individual cell extend across several input/output compartments. (B) For comparison, the afferent/efferent relationships of the CM-Pf complex in the thalamus of an anthropoid primate (squirrel monkey, *Saimiri sciureus*) are shown following the same graphic conventions. This representation is based on Sadikot and Rymar (2009), with additional squirrel and macaque monkey data from Mufson and Mesulam (1984), Miyata and Sasaki (1984), Goldman-Rakic and Porrino (1985), Yeterian and Pandya (1988), Nakano et al. (1993), Stepniewska et al. (1994, 2007), Rouiller et al. (1998), Cavada et al. (2000), François et al. (2002), Sidibé et al. (2002), Kultas-Ilinsky et al. (2003), and Hsu and Price (2007). We labeled the rodent entopeduncular nucleus as “GPi” to make similarities/differences between rodents and primates more readily perceptible. Scale bars: 500 μm. A24, A25, A32, anterior cingulate cortices; bf, barrel field; Cd, caudate; CPu, caudate-putamen; DCNs, deep cerebellar nuclei; FEF, frontal eye field; FrA, frontal association cortex; GPe, external globus pallidus; GPi, internal globus pallidus; Ins, insular cortex; LC, locus coeruleus; NAc, nucleus accumbens; Orb, orbital cortex; PAG, periaqueductal gray matter; PBNs, parabrachial nuclei; PFC, prefrontal cortex; PFR, pontine reticular formation; PPN, pedunculopontine nucleus; Pu, putamen; SNr-l, substantia nigra reticular part, lateral; SNr-m, substantia nigra reticular part, medial; STN, subthalamic nucleus; TRN, thalamic reticular nucleus; VTA, ventral tegmental area; ZI, zona incerta.

Each cortical area selectively targets a specific Pf portion along the whole anteroposterior length of Pf. Moreover, these portions are consistently related to specific sensory-motor or cognitive subsystems. For example, the corticothalamic inputs from regions of areas M1, M2, or S1 associated with the movement/sensation of mouth, forelimb, or hindlimb/trunk (Chapin and Lin, 1984; Tennant et al., 2011; Foster et al., 2021) converge each in a specific Pf portion (Figure 13). Similarly, medial frontal and cingulate areas innervate a dorsal zone of medial Pf, while lateral orbital and insular areas target a more ventral zone. The dendrites of Pf neurons are long, and thus may extend across more than one of these area-specific subdomains (Figure 15A), suggesting that they may integrate corticofugal signal flows related to different body parts.

In register with some early retrograde tracing reports in primates, cats, and rats (Catsman-Berrevoets and Kuypers, 1978; Royce, 1983; Cornwall and Phillipson, 1988), we show here that about 45% of corticothalamic projections to mouse Pf originate from cortical L5b. This proportion is far above that reported in any other nucleus of the rodent thalamus (Killackey and Sherman, 2003; Jones, 2007; Casas-Torremocha et al., 2022). Recent single-cell reconstruction studies (Economo et al., 2018) have shown that the thalamus-projecting mouse L5b pyramidal cells from motor cortex innervate the thalamus, SC, and pons, but do not reach more caudal brainstem levels; hence, they may be mainly involved in premotor functions. In other thalamic nuclei, the L5b axons have been shown to terminate as large boutons on proximal dendrites that powerfully drive thalamic cell firing mainly through ionotropic receptors (Groh et al., 2014; Sherman, 2016). Future studies should explore the synaptic organization and receptor mechanisms involved in the interactions of L5b corticothalamic axons and Pf cells.

Notably, we show here a relatively small, but consistent L5b projection to Pf from the contralateral hemisphere (Figures 10C,E, 11B,C; Supplementary Figure S5). This projection arises from the motor, insular, orbital, cingulate, and somatosensory areas. To our knowledge, contralateral L5b corticothalamic projections had not been previously reported.

### Superior colliculus inputs

We show that the SC is the main subcortical source of excitatory afferents to mouse Pf in terms of the number of projection neurons involved. This projection arises mostly from large multipolar neurons in the lateral half of the intermediate and deep layers of the ipsilateral SC, with a small contralateral contribution. There is some spatial segregation in these inputs, as projections to lateral Pf arise from the most lateral zone of these SC layers, which are known to be involved in somatosensory/motor integration, while projections to the medial/dorsal portion of Pf arise from a more medial zone, associated with oculomotor integration (Figures 14I–L; Benavidez et al., 2021). Multipolar neurons in these SC layers originate tectal projections to the brainstem and spinal cord (Krout et al., 2001; Benavidez et al., 2021).

Through the projections to Pf, multimodal sensory SC signals may thus influence the whole dorsal striatum in di-synaptic fashion (McHaffie et al., 2005; Redgrave et al., 2010; Alloway et al., 2017). Studies in primates have shown that CM-Pf neurons respond to salient sensory events to modulate premotor processes, such as behavioral and attentional switching and action biasing (Matsumoto et al., 2001; Minamimoto and Kimura, 2002; Minamimoto et al., 2005, 2009). In rodents, SC-thalamus inputs have been shown to carry fast, low-resolution visual signals that are important for innate species-specific responses to predators (Wei et al., 2015).

Overall, our data show that cortical L5b and SC axon terminals overlap extensively within Pf. Future studies should elucidate if these two excitatory input systems converge on the same postsynaptic cells and, if so, how their signals are computed by Pf cells (Groh et al., 2014; Acsády, 2022).

### Inhibitory basal ganglia, TRN, and brainstem inputs

The mouse Pf lacks local inhibitory interneurons (Jones, 2007). However, it receives inhibitory connections from several sources. First, and consistent with previous studies (Deniau et al., 1992; Deniau and Chevalier, 1992; Mengual et al., 1999; Kha et al., 2000, 2001; Mastro et al., 2014; Foster et al., 2021; Lilascharoen et al., 2021), we show here that the basal ganglia output nuclei (i.e., EPN and SNr) are a substantial source of input to Pf. These GABAergic projections are known to be collateral branches of axons directed to brainstem motor nuclei (Cebrián et al., 2005). They are believed to provide powerful, temporally precise, and focal inhibition, able to impact the spike timing of thalamic cells (Halassa and Acsády, 2016).

Second, as for all other thalamic nuclei, the TRN provides inhibitory input to Pf. Intriguingly, despite the caudal location of Pf within the thalamus, the TRN neurons that innervate it are located in a rostral and ventral part of TRN. The same TRN zone has been reported to project back to Pf in rats (Kolmac and Mitrofanis, 1997) and to be selectively targeted by the axons from other motor thalamic nuclei, as well as by corticothalamic axons from the motor cortex (Hádinger et al., 2023).

A third source of inhibitory input reportedly arises from glycinergic neurons in the oral pontine reticular formation (Giber et al., 2015). We observed heavy GlyT2 neuropil immunostaining in the dorsomedial Pf, but the retrogradely-labeled reticular formation neurons in our experiments were located mainly in the mesencephalon and relatively scarce in the oral pons.

### Pf output pathways to the dorsal striatum

The dorsal striatum is, by far, the main target of Pf axons. As a whole, the Pf axons arborize in all regions of the dorsal striatum in a patchy, discontinuous fashion. Our anterograde experiments did not label substantial Pf projections to the ventral striatum (nucleus accumbens and olfactory tubercle), a region that receives its thalamic input mainly from the paraventricular nucleus, as well as from Pf neurons situated medially to the retroflex bundle (a zone not sampled in our dataset; Berendse and Groenewegen, 1990; Erro et al., 2002; Foster et al., 2021).

A focal BDA injection in Pf invariably produced multiple separate, dorsoventrally elongated terminal arborization patches in the striatum, consistent with the reported morphology of individual thalamostriatal Pf axons (Deschênes et al., 1995, 1996; Parent and Parent, 2005). These arborizations were selectively located in the striatal matrix compartment; however, it is unclear whether they reach the whole extent of this compartment, or just a part of it. In primates, the matrix zones targeted by CM-Pf axons have been shown to be different from the ones originating the projection to the globus pallidus (Sidibé and Smith, 1996; see also Giménez-Amaya and Graybiel, 1991).

The Pf projection to the dorsal striatum is topographic (Figure 15A). A dorsal-to-ventral gradient is clearly detectable in the origin of lateral Pf pathways to the sensorimotor/lateral striatum (Figures 4–7). This segregation was recently linked to the somatotopic pattern implied by the topography of corticostriatal projections from motor and sensory areas (Hintiryan et al., 2016; Foster et al., 2021). For example, the ventrolateral part of Pf innervates striatal and cortical regions involved in the motor control of the mouth and face, whereas more dorsal portions of lateral Pf innervate regions that are associated with the motor control of the limbs and trunk (Carelli and West, 1991; Iwai et al., 2015; Hintiryan et al., 2016). The medial Pf neurons innervate the anterior and medial portions of the dorsal striatum (Figure 15A); these portions are also innervated by orbital frontal, insular and cingulate areas involved in visceral sensation/regulation and oculomotor adjustments (Vogt, 2019; Benavidez et al., 2021; Foster et al., 2021; Le Merre et al., 2021).

### Pf output pathways to the subthalamic nucleus

Unlike other thalamic nuclei, Pf provides a direct innervation to STN. These connections are established through collateral branches of the axons that simultaneously innervate the striatum and cortex, but only a fraction (about 20%) of the Pf axons reportedly possess this collateral branch in rats (Deschênes et al., 1996; Unzai et al., 2017). BDA injections in different portions of Pf suggest the existence of relatively independent projection channels (Figure 8; Supplementary Figure S3; see also Bevan et al., 1995; Kita et al., 2016). By monosynaptically eliciting the firing of STN cells (Mouroux et al., 1995), Pf axons can thus act on yet another node of the basal ganglia network (Lanciego et al., 2004; Watson et al., 2021; Fallon et al., 2023).

### Pf output pathways to the cerebral cortex

Consistent with observations in some previous rat studies (Herkenham, 1986; Deschênes et al., 1996; Unzai et al., 2017), our data demonstrate that Pf axon arborizations in the cortex are always loose and poorly branched, and they do not form focal plexuses into particular columns or layers. The Pf axons reach all cortical layers, with a slightly higher prevalence in the infragranular layers. The scarcity of the Pf thalamocortical projection is in striking contrast with the massive corticothalamic L5b and L6 projections that this nucleus receives (Figures 10D, 13). Overall, the Pf thalamocortical axon morphology is quite unlike that of most other thalamocortical projections, and reminiscent of the immature morphologies observed in early postnatal axons (Galazo et al., 2008).

The Pf thalamocortical axons are preferentially directed to the motor and premotor (M1, M2, FrA), orbital and agranular insular areas. Additional projections reach somatic sensory and anterior cingulate cortices. As a rule, the thalamocortical projections originated in a particular portion of Pf terminate in the same areas that originate the corticothalamic projections to that portion (Figure 15A). Moreover, Pf thalamocortical projections systematically target the same cortical territory that innervates the striatal sector where the thalamostriatal axons from that Pf region terminate. This wiring logic may be viewed as evidence that Pf axons link functionally related cortical and striatal nodes of specific motor and premotor subnetworks (Foster et al., 2021). Our results, however, show that the most robust and spatially convergent pathways out of Pf are those directed towards the striatum, not the cortex (Supplementary Figure S2). Hence, the impact of Pf axons on postsynaptic cell firing may be markedly different in the two structures. In this regard, we find it remarkable the absence in the literature of reports about changes in cortical unit activity evoked by stimulation of Pf, in contrast with the numerous reports of changes caused in the activity of striatal neurons (Baldi et al., 1995; Consolo et al., 1996a,b; Nanda et al., 2009; Ding et al., 2010; Ellender et al., 2013; Alloway et al., 2014; Arias-García et al., 2018; Mandelbaum et al., 2019; Lee et al., 2020).

### Comparison of Pf axonal varicosity sizes in different target structures

In glutamatergic axons, varicosity size correlates with the strength and dynamic properties of synapses (Rovó et al., 2012; Groh et al., 2014; Sherman, 2016; Rodriguez-Moreno et al., 2020). Here, we measured and compared the size of labeled Pf axon varicosities in the striatum, cortex and STN and found that it is similar in the three structures, albeit the varicosities in the striatum are slightly larger (Figure 8A). Within the striatum, we found a slight difference in the size of varicosities between the central focus and the periphery of the arborizations labeled by BDA injections in specific Pf subregions.

### Comparison of mouse Pf and primate CM-Pf input–output motifs

Our data show different input–output motifs for lateral/ventral vs. medial/dorsal portions of the rodent Pf nucleus. These differences show clear parallels with the connections described for the primate CM and Pf nuclei (Figure 15; Smith et al., 2004; Galvan and Smith, 2011). In addition, the staining patterns for CB, AChE and GlyT2 in these nuclei are strikingly similar in both phyla (Figures 1, 2; Supplementary Figure S6; Sadikot et al., 1992; Paxinos et al., 2012; Giber et al., 2015). For clarity and based on the anatomical evidence now available, we suggest that the name “Parafascicular” should be applied only to the medial/dorsal portion of the mouse cell mass classically named Pf; in turn, the large lateral/ventral portion of this cell mass should be simply referred to as “Centromedian” (CM), as in primates (Figure 15A).

In summary, our study reveals a highly conserved basic plan of CM and Pf input–output motifs in both rodents and primates. Interestingly, the limited single-cell morphology evidence available suggests that even the layout of individual axons is fundamentally similar between the two phyla, albeit with a greater specialization/diversity in the axon branching patterns of primate CM-Pf neurons (Deschênes et al., 1996; Parent and Parent, 2005). These observations indicate that research in mouse CM-Pf cells and circuits may illuminate the role of this nucleus in motor function and disease, not only in rodents but also in primates.

## Data availability statement

The original contributions presented in the study are included in the article/Supplementary material, further inquiries can be directed to the corresponding author.

## Ethics statement

The animal study was approved by Comité de Ética de la Investigación, Universidad Autonoma de Madrid under the Madrid Regional Government agency protocol (PROEX 179.3/21). The study was conducted in accordance with the local legislation and institutional requirements.

## Author contributions

EG-M: Methodology, Visualization, Data curation, Formal analysis, Investigation, Writing – original draft. CA-M: Formal analysis, Investigation, Writing – review & editing. LS: Formal analysis, Investigation, Writing – review & editing, Conceptualization, Data curation, Funding acquisition, Visualization, Writing – original draft. FC: Conceptualization, Funding acquisition, Investigation, Visualization, Writing – review & editing, Methodology, Project administration, Supervision.
