# Supplementary material for: Micropopulation mapping of the mouse parafascicular nucleus connections reveals diverse input–output motifs
Source: Front Neuroanat. 2024 Jan 8;17:1305500. doi: 10.3389/fnana.2023.1305500 (PMC10800635; doi:10.3389/fnana.2023.1305500)
Supplement: Supplementary file 1 [file Data_Sheet_1.PDF]

Supplementary Materials

Micropopulation mapping of the mouse parafascicular nucleus reveals diverse input-output motifs

Gonzalo-Martín, E.; Alonso-Martínez, C.; Prensa, L.; Clascá, F.

Frontiers in Neuroanatomy 2023

**Target**	**Host animal**	**Reference**	**Source**	**Dilution**
Mu-opioid receptor (MOR)	Rabbit	AB1580-I	Merck	1:1500
Cholera Toxin B subunit (CTb)	Rabbit	C3062	Sigma-Aldrich	1:500
Glycine Transporter Type-2 (GlyT2)	Guinea pig	272 004	Synaptic Systems	1:2000
Calbindin (CB)	Mouse	C9848	Sigma-Aldrich	1:100

Supplementary Materials Table 1. Primary antiserums used in this study.

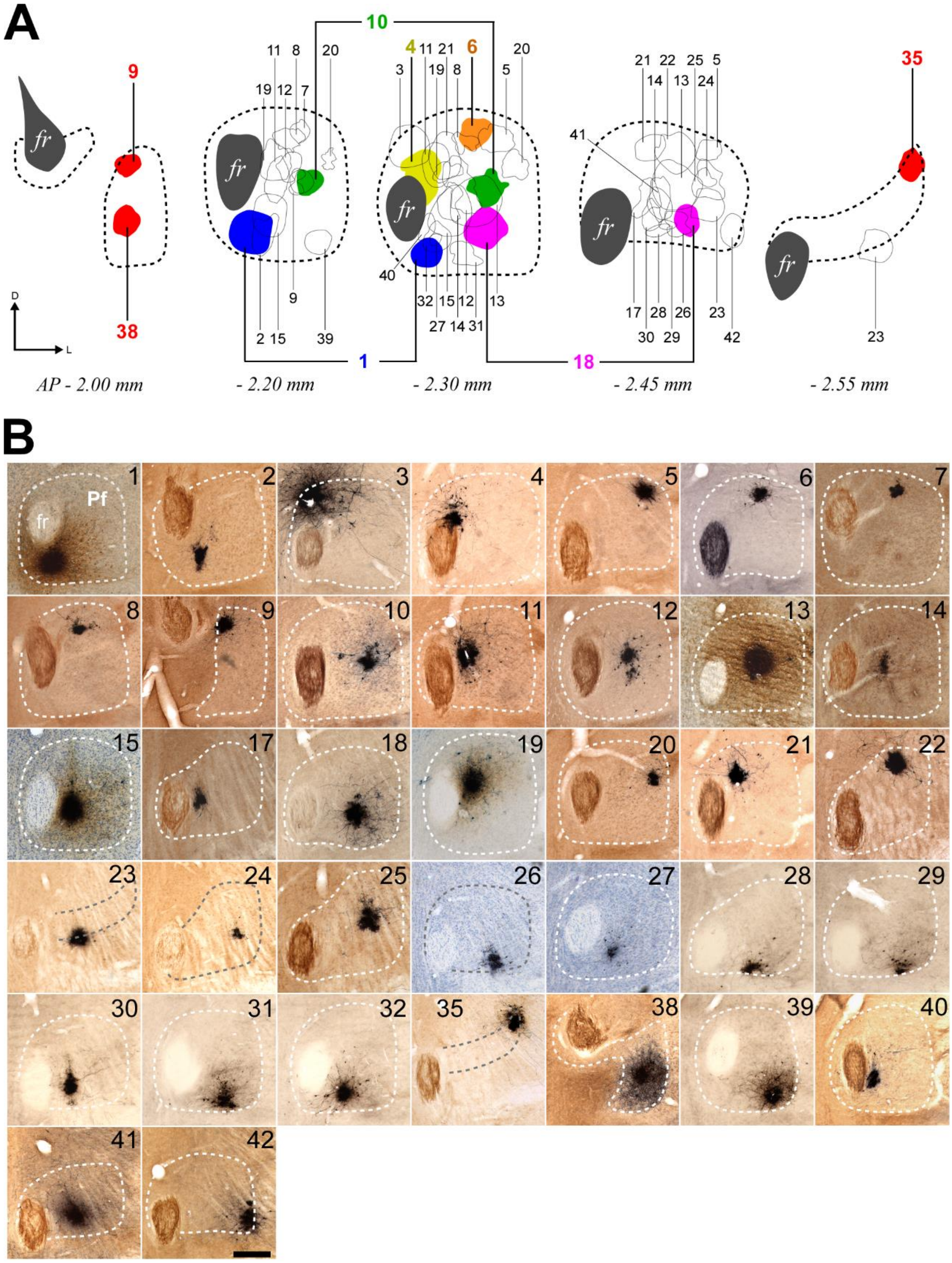

Supplementary Materials Figure 1. BDA and BDA+CTb experiments used in the present study. A) Diagram showing all BDA deposits analyzed. The extent of each injection is represented by line contours on coronal views of Pf section diagrams from rostral (left) to caudal (right); compare with Figure 1. Representative cases that are described and illustrated in subsequent figures are highlighted here in different colors. **B)** Photomicrographs showing all valid BDA and BDA+CTb injection sites analyzed. Values in mm under the drawings indicate the Bregma level (B). Scale bar: 200 µm.

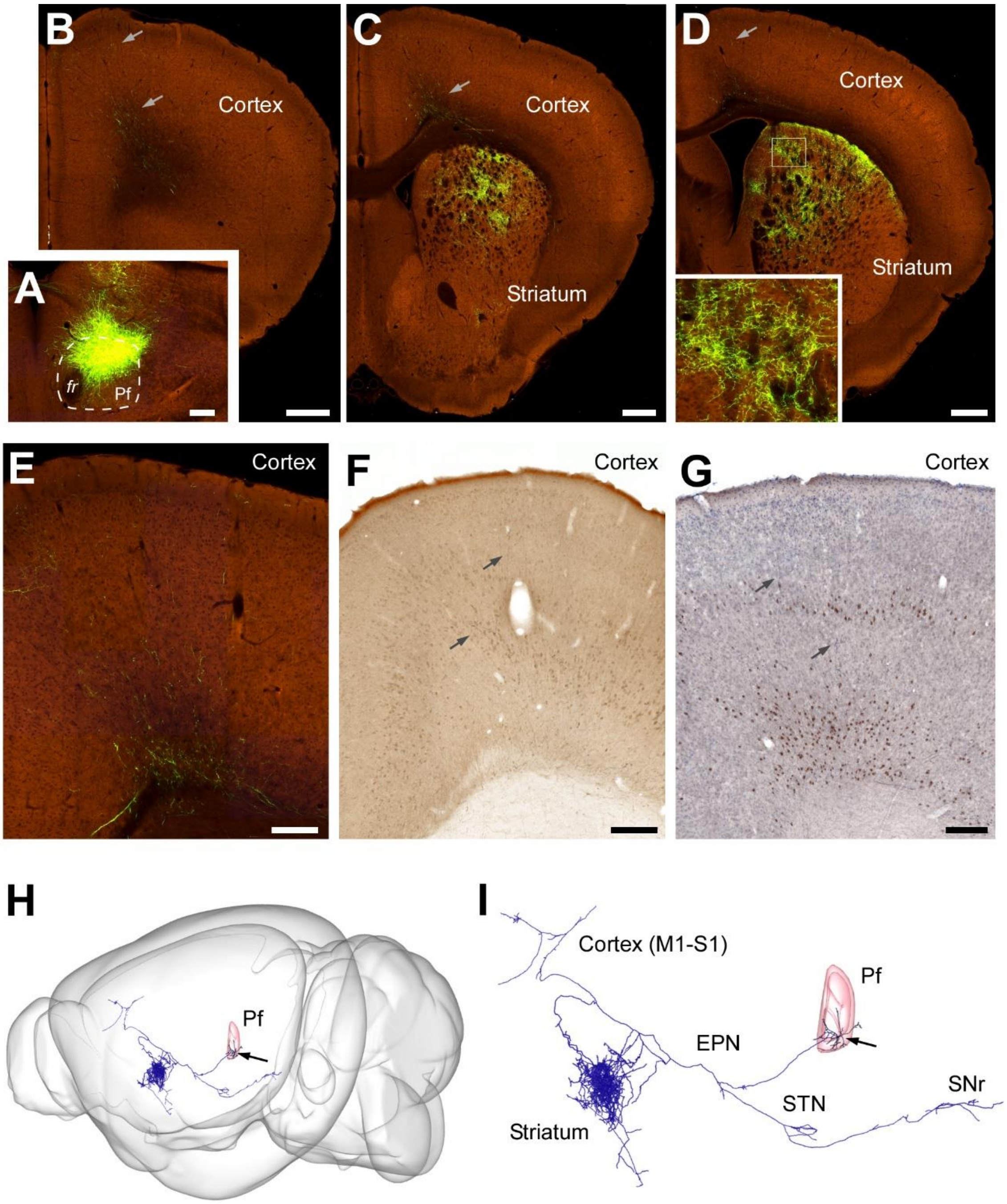

Supplementary Materials Figure 2. Highly unequal anatomical weight of the thalamostriatal vs thalamocortical projection from Pf neurons. (A) Two-photon tomography image sample from an experiment in which AAV-GFP vectors were injected in dorsal Pf (Allen Institute Mouse Brain Connectivity; experiment ID: 300642574). The center of the injection site in Pf is shown. **(B-D)** Three coronal section images of the frontal portion of the cerebral hemisphere in the same experiment. Note the virtual absence of labeled axons in the cortex (indicated by arrows), and the massive, patchy fluorescent labeling of thalamostriatal axons in striatum. Inset in "D" shows a high magnification of the richly branched thalamostriatal axon arborizations. **(E-G)** Similar coronal section samples of the motor cortex are compared among experiments in which Pf was injected with either AAV-GFP (panel "E"), BDA (panel "F"), or BDA+CTb (panel "G"). In all cases, the labeled thalamocortical axons (arrows in "F" and "G") were very scant and sparse. **(H-I)** A representative single-cell morphology of an individually

labeled Pf neuron (image taken from the Janelia Research Campus Mouselight Database; Winnubst et al., 2019; https://ml-neuronbrowser.janelia.org/; neuron ID: AA1439). The cell body and dendrites are indicated by an arrow. The volume of Pf is displayed as a transparent-pink shape. Observe that this cell axon leaves most of its terminal branches in the striatum, while its thalamocortical branch is extremely simple. Scale bars: 250 μm (A), 500 μm (B, C, D), or 200 μm (E, F, G).

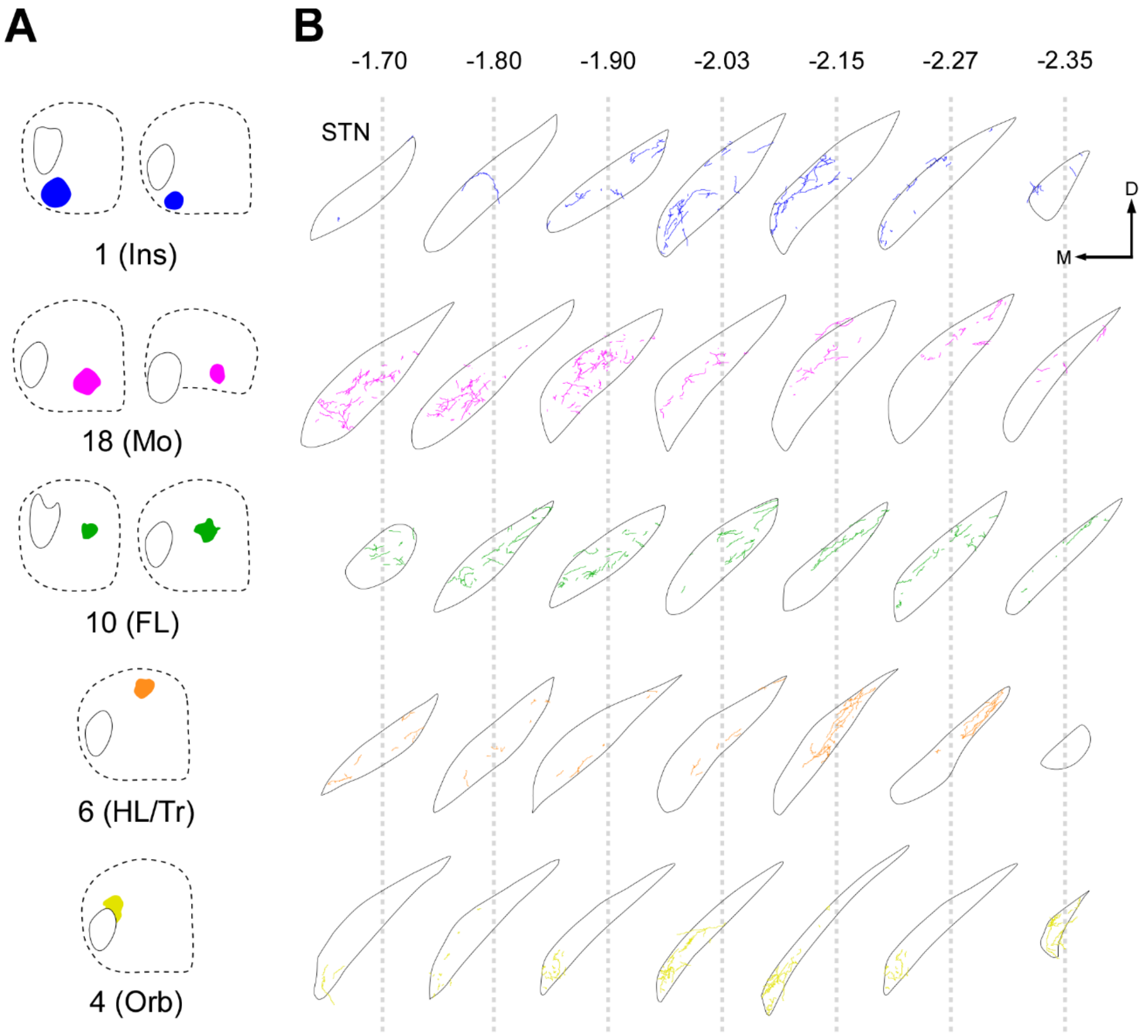

Supplementary Materials Figure 3. Projection to the STN from neuron micropopulations located in different portions of Pf. A) Location within Pf of the five BDA deposits used for this study, adjusted to the Paxinos and Franklin atlas sections (2019). The five experiments represented here are the same ones that were illustrated as representative cases in sections 2, 3 and 4 of the Results (Figures 3-9). **B)** Arborization within the STN of axons arising from each of the five BDA deposits. STN contours and Pf axons were traced in all cases using Neurolucida® software tools on live sections. The tracings represent the whole rostrocaudal extent of the STN.

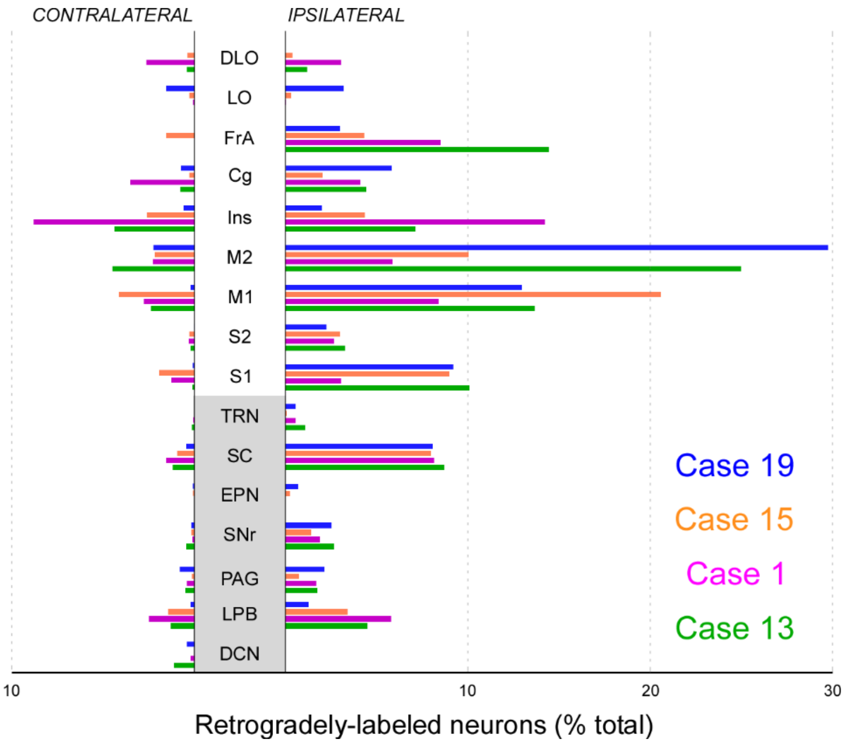

Supplementary figure 4. Quantitative analysis of the afferents innervating different portions of the mouse Pf. Relative percentages of retrogradely labeled neurons observed throughout the brain after CTb deposits into four different portions of mouse Pf. Neurons in the ipsilateral (right) and contralateral (left) hemispheres are separated for each structure. These four experiments are the same that are averaged in Figure 11.

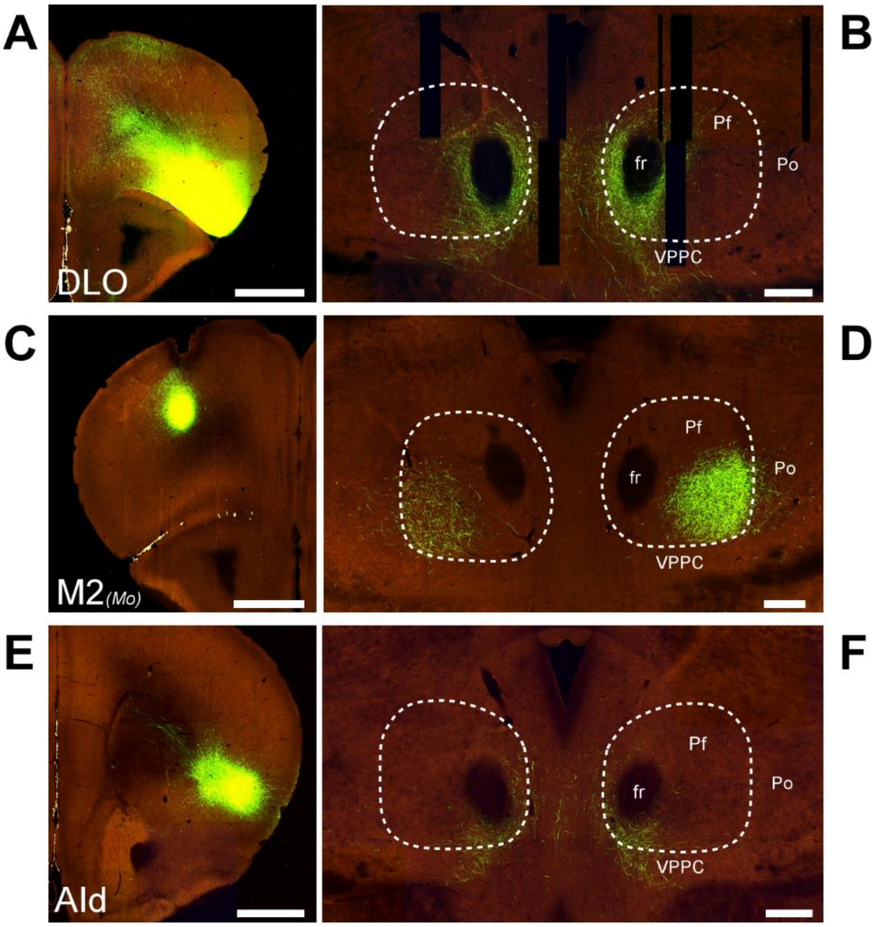

Supplementary figure 5. Different cerebral cortex areas project bilaterally to mouse Pf, keeping the same topographic arrangement. Two-photon tomography image samples from experiments in which AAV vectors able to drive the expression of high levels of fluorescent protein were injected in different areas. **(A-B):** Corticothalamic projection from DLO. **(C-D):** Projection from rostral M2 (putative mouth zone). **(E-F):** Projection from AId. The three experiments illustrated here are represented also in Figure 13. Scale bars: 1 mm (A, C, E) or 250 mm (B, D, F). Abbreviations: AId: agranular insular cortex, dorsal part; DLO: dorsolateral orbital cortex; fr: fasciculus retroflexus; M2: secondary motor cortex; Pf: parafascicular nucleus; Po: posterior nucleus; VPPC: ventroposterior nucleus, parvicellular part.

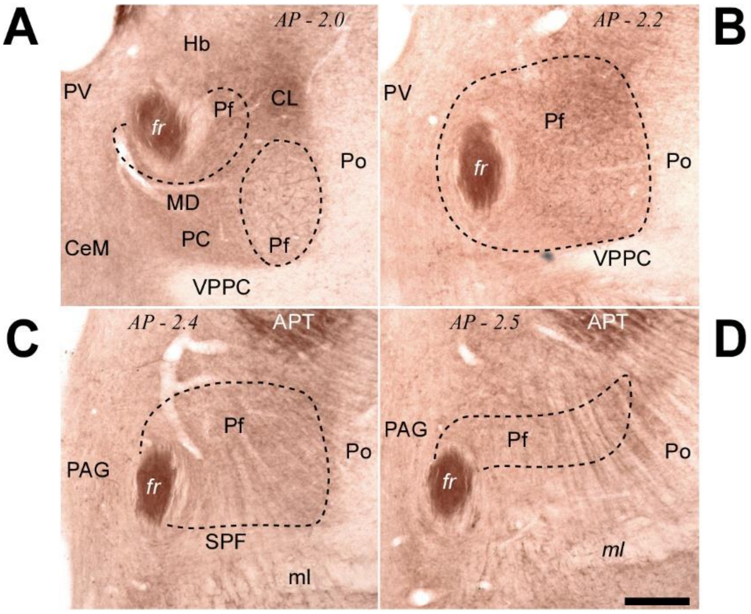

Supplementary Materials Figure 6. Acetylcholinesterase (AChE) staining throughout the Pf, from rostral (A) to caudal (D). Scale bar: 250 µm.
